# Immune Regulatory Neural Stem/Precursor Cells Protect from Central Nervous System Autoimmunity by Restraining Dendritic Cell Function

**DOI:** 10.1371/journal.pone.0005959

**Published:** 2009-06-19

**Authors:** Stefano Pluchino, Lucia Zanotti, Elena Brambilla, Patrizia Rovere-Querini, Annalisa Capobianco, Clara Alfaro-Cervello, Giuliana Salani, Chiara Cossetti, Giovanna Borsellino, Luca Battistini, Maurilio Ponzoni, Claudio Doglioni, Jose Manuel Garcia-Verdugo, Giancarlo Comi, Angelo A. Manfredi, Gianvito Martino

**Affiliations:** 1 Neuroimmunology Unit, San Raffaele Scientific Institute and Università Vita–Salute, Milan, Italy; 2 DIBIT II and Institute of Experimental Neurology (InSpe), San Raffaele Scientific Institute and Università Vita–Salute, Milan, Italy; 3 Department of Neurology and Neurophysiology, San Raffaele Scientific Institute and Università Vita–Salute, Milan, Italy; 4 Clinical Immunology Unit, San Raffaele Scientific Institute and Università Vita–Salute, Milan, Italy; 5 Pathology Unit, San Raffaele Scientific Institute and Università Vita–Salute, Milan, Italy; 6 Neuroimmunology Unit, European Brain Research Institute, Santa Lucia Foundation, Rome, Italy; 7 Department Comparative Neurobiology, Instituto Cavanilles, University of Valencia, Valencia, Spain; 8 Department of Cellular Therapy, Centro de Investigación Príncipe Felipe, Valencia, Spain; 9 Instituto de Ciências Biomedicas Abel Salazar (ICBAS), Universidade do Porto, Porto, Portugal; Julius-Maximilians-Universität Würzburg, Germany

## Abstract

**Background:**

The systemic injection of neural stem/precursor cells (NPCs) provides remarkable amelioration of the clinico-pathological features of experimental autoimmune encephalomyelitis (EAE). This is dependent on the capacity of transplanted NPCs to engage concurrent mechanisms of action within specific microenvironments in vivo. Among a wide range of therapeutic actions alternative to cell replacement, neuroprotective and immune modulatory capacities of transplanted NPCs have been described. However, lacking is a detailed understanding of the mechanisms by which NPCs exert their therapeutic plasticity. This study was designed to identify the first candidate that exemplifies and sustains the immune modulatory capacity of transplanted NPCs.

**Methodology/Principal Findings:**

To achieve the exclusive targeting of the peripheral immune system, SJL mice with PLP-induced EAE were injected subcutaneously with NPCs and the treatment commenced prior to disease onset. NPC-injected EAE mice showed significant clinical improvement, as compared to controls. Exogenous NPCs lacking the expression of major neural antigens were reliably (and for long-term) found at the level of draining lymph nodes, while establishing sophisticated anatomical interactions with lymph node cells. Importantly, injected NPCs were never found in organs other than lymph nodes, including the brain and the spinal cord. Draining lymph nodes from transplanted mice showed focal up-regulation of major developmental stem cell regulators, such as BMP-4, Noggin and Sonic hedgehog. In lymph nodes, injected NPCs hampered the activation of myeloid dendritic cells (DCs) and steadily restrained the expansion of antigen-specific encephalitogenic T cells. Both ex vivo and in vitro experiments identified a novel highly NPC-specific–BMP-4-dependent–mechanism hindering the DC maturation.

**Conclusion/Significance:**

The study described herein, identifies the first member of the TGF β/BMP family of stem cell regulators as a novel tolerogenic factor released by NPCs. Full exploitation of this pathway as an efficient tool for vaccination therapy in autoimmune inflammatory conditions is underway.

## Introduction

Spontaneous neural tissue repair may occur in acute and/or chronic inflammatory and degenerative disorders of the nervous system such as multiple sclerosis (MS). However, this process is not robust to promote a full functional and stable recovery of the nervous system architecture [1]. Recent advances in (stem) cell biology have raised great expectations that diseases and injuries of the central nervous system (CNS) may be ameliorated by the development and delivery of cell therapies. Though, most (if not all) of the experimental cell therapies described, injecting neural lineage-committed progenitors, have failed to foster substantial tissue repair in disease models where the anatomical and functional damage is widespread and an inflamed and/or degenerative microenvironment co-exists [2]. In contrast, the systemic injection of somatic, and more recently embryonic stem (ES) cell-derived, neural stem/precursor cells (NPCs) has provided remarkable amelioration of the clinico-pathological features of rodents with acute, chronic and relapsing experimental autoimmune encephalomyelitis (EAE), the animal model of MS [3], [4], [5], [6].

This phenomenon has been shown to be dependent on the capacity of transplanted NPCs to engage multiple mechanisms of action within specific microenvironments in vivo [7]. Among a wide range of potential therapeutic actions, and, in addition to the (expected) cell replacement [3], remarkable neuroprotective and immune modulatory capacities have been described for transplanted NPCs within specific CNS [3], [4], [5], [6] as compared to non-CNS areas [8]. As such, we and others have provided considerable proof that NPC-mediated bystander effects may take place both in the CNS, at the level of the atypical perivascular niches [6], as well as in secondary lymphoid organs, such as the lymph nodes [8] or the spleen [9]. Nonetheless, following the first report that membrane-bound Fas/CD90 ligands (e.g., Apo3L, TRAIL and FasL) were regulating part of the NPC-mediated suppressive effect on encephalitogenic T lymphocytes in the CNS [6], other groups have generated data albeit indirectly that describe the mechanisms responsible for this peculiar somatic stem cell function. In general, this has been supported in studies that utilized in vitro immune cell/NPC co-cultures [4], [5], [10], although these studies have provided evidence describing only the short-term in vivo persistence of transplanted NPCs into peripheral (non-CNS) bodily organs [8], [9]. Concurrently, recent reports have begun to elucidate paracrine factors that are responsible for mediating the immune suppressive *vs* pro-survival capacity of other somatic stem cell sources; these include chemokines and inducible nitric oxide (iNOS) [11] and more recently stanniocalcin-1 (STC-1), a peptide hormone that modulates mineral metabolism [12]. Importantly though, the detailed molecular and cellular mechanism(s) responsible for sustaining the multifaceted therapeutic plasticity exhibited by NPCs in vivo remain far from being fully elucidated, characterized and described.

Herein we report the capacity of NPCs to target, and synergize with immune cells in secondary lymphoid organs (e.g., draning lymph nodes), and have demonstrated this by utilizing a highly peculiar protocol of therapeutic passive NPC vaccination in mice affected by experimental chronic-recurrent autoimmune CNS inflammation. Subcutaneously (s.c.)-injected NPCs accumulate and survive over two months within draining lymph nodes, but not in the CNS, where they stably modify the perivascular lymph node microenvironment. Within this context, surviving NPCs hamper the activation of myeloid dendritic cells (DC) via the release of major developmental stem cell regulators, including the morphogens bone morphogenetic protein (BMP)-4 and sonic hedgehog (Shh), the extracellular matrix protein tenascin C, and the BMP antagonist Noggin.

Nonetheless, we identify a novel BMP-4-dependent mechanism hindering the DC maturation, both in vivo and in vitro. This BMP-dependent effect is highly specific for immune regulatory NPCs, and, in turn, lead to the steady restraint of the expansion of antigen-specific (encephalitogenic) T cells.

## Results

### Protection of EAE mice upon accumulation of s.c.-injected NPCs into draining lymph nodes

SJL mice suffering from relapsing-remitting experimental autoimmune encephalomyelitis (R-EAE), a model of chronic-relapsing autoimmune CNS inflammation leading to demyelination and axonal loss [6], were injected s.c. into the flanks with subventricular zone (SVZ)-derived syngenic adult NPCs (1.0×10^6^ cells per mouse). Green fluorescent protein (GFP)^+^ NPCs were injected at either 3 and 10 days post immunization (dpi) with the myelin autoantigen proteolipid protein (PLP)139–151, or at 10 dpi only. Regardless of the timing or number of injections, both groups of NPC-injected R-EAE mice showed significant clinical improvement, as compared to sham-treated controls. Further, mice injected at 10 dpi only, showed a significant delay of disease onset (p≤0.005, vs. sham-treated controls). At the end of the follow up period (75 dpi), both groups of NPC-treated mice showed significantly lower R-EAE cumulative score, fewer clinical relapses, and lower burden of spinal cord inflammation (from 46 to 64% reduction), demyelination (from 84 to 98% reduction) and axonal damage (from 89 to 93% reduction), as compared to sham-treated controls. Finally, *delayed* injection (injected at 30 dpi) of live NPCs or paraformaldehyde (PFA)-fixed NPCs (injected at 10 dpi) did not produce any detectable clinical improvement, as compared to sham-treated controls (Figure S1). The clinico-pathological features of R-EAE mice injected s.c. with different NPC types are summarised in Table 1 and Figure S1.

**Table 1:** Clinico-pathological features of R-EAE mice injected s.c. with NPCs.

Treatment	Treatment schedule	No. of mice	Disease onset (dpi)‡	Maximum clinical score‡	Cumulative disease score† ‡	Number of relapses	Inflammatory infiltrates§ * (no./mm^2^)	Demyelination§ ‡ (%/mm^2^)	Axonal loss§ ‡ (%/mm^2^)
Sham	3 and 10 dpi	10	15.8±0.9	2.35±0.07	110.35±7.7	2.14±0.1	3.3±0.6	1.17±0.3	0.95±0.2
NPCs	3 and 10 dpi	15	15.6±1	1.75±0.2*	83±12.7*	1.5±0.2*	1.2±0.3**	0.19±0.09**	0.1±0.04**
NPCs	10 dpi	15	21.2±1.5**	1.87±0.1*	69.9±9.5**	1.2±0.1**	1.8±0.4*	0.02±0.01**	0.07±0.02**

‡ Data are mean numbers (±SEM) from a total of n = 2 independent experiments. dpi, days post-immunization.

† The cumulative score represents the summation of each single score recorded for each mouse from the day of immunization (day 0) to day of sacrifice (75 dpi).

§ Inflammatory infiltrates, demyelination and axonal loss have been quantified at sacrifice (75 dpi) on an average of n≥20 spinal cord sections per mouse from a total of n = 3 mice per group.

* p≤0.05 when compared with sham-treated controls.

** p≤0.005 when compared with sham-treated controls.

**Figure 1:**
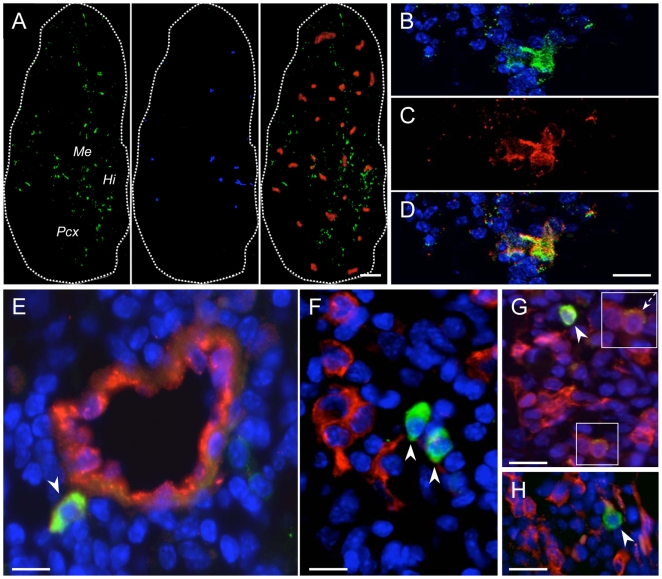
S.c.-injected NPCs accumulate and persist in secondary lymphoid organs from R-EAE mice while lacking the expression of major antigens of the neural lineage. A, Sagittal reconstruction of a representative axillary lymph node from a R-EAE mouse injected s.c. with syngeneic NPCs. GFP^+^ NPCs (green) are detected throughout the entire lymph node tissue although they predominantly accumulate as focal clusters in the hilum (Hi), medulla (Me), and paracortex (Pcx). Some of the NPCs are in close contact with CD11b^+^ macrophages (red). Twenty % of NPCs express the early neuronal differentiation marker doublecortin (DCX) (blue). Scale bar: 300 µm. B–D, Occasionally, GFP^+^ NPCs (B) expressing the neural cell marker nestin (C) are detected in perivascular lymph node areas. The panel in D is a merged image of the pictures in B and C; E, Representative image of a cervical lymph node where NPCs (green, arrowhead) are found to establish close anatomical interaction(s) with von Willebrand factor antigen-expressing endothelial cells (red); F, Confocal microscopy image of a para-aortic lymph node where NPCs (green, arrowheads) are found in close cell-to-cell contact with CD11c^+^ DCs (red). G and H, Axillary lymph node sections showing persistence of NPCs (green, arrowheads) in areas colonized by F4/80^+^ phagocytes (G, red) and MHC class-II^+^ immune cells (H, red). Occasionally, F4/80^+^ cells being immunoreactive also for GFP (G, dashed arrow in the boxed area) are identified. NPCs are in green in A–H. Nuclei in B, D and E–H are counterstained with DAPI. Scale bars in B–H: 40 µm. Data refer to R-EAE mice injected s.c. with NPCs at 3 and 10 dpi and sacrificed 72 days after cell injection.

To assess quantity, morphology and distribution of s.c.-injected NPCs, a detailed histological analysis was performed at 75 dpi in both the CNS (e.g., brain and spinal cord) and peripheral tissues such as spleen, liver, kidneys and draining lymph nodes from transplanted R-EAE mice. Quite distinct from what occurs upon injection of NPCs either intravenously or intrathecally into EAE rodents after disease onset [3], [6], [13], we did not find s.c.-injected GFP^+^ cells in the brain, spinal cord, liver, spleen and kidneys. On the other hand, GFP^+^ NPCs were consistently found at both early (e.g., 2 weeks after cell injection) as well as late (e.g., 65–72 days after cell injection) time points in the draining lymph nodes of all NPC-injected R-EAE mice (following both treatment schedules). The number of GFP^+^ cells accumulating and persisting for at 65–72 days after cell injection within draining lymph nodes varied only moderately from case to case. The highest number of GFP^+^ NPCs was observed in axillary and cervical lymph nodes proximal to the cell injection site(s). While the lymph node architecture was preserved and low-power examination did not disclose any alteration, NPCs predominantly accumulated as focal clusters around blood vessels of the hilum and medullary/paracortical areas (Figure 1A). The great majority of s.c.-injected NPCs survived long term (greater than 2 months after transplantation) within lymph nodes, while lacking the expression of major antigens of the neural lineage, such as polysialylated neural cell adhesion molecule (PSA-NCAM), class III β–Tubulin, neuronal nucleus (NeuN), NG-2, glial fibrillary acidic protein (GFAP) and platelet-derived growth factors receptor (PDGFr)α. However, 20% of the GFP^+^ NPCs expressed the early neuronal differentiation marker doublecortin (DCX) (Figure 1A and Figure S2), while10% of GFP^+^ NPCs were immune reactive for the neural cell marker nestin in lymph nodes (Figure 1B–D).

### Interaction(s) of NPCs and lymph node cells at the level of perivascular areas

In lymph nodes, NPCs established anatomical interaction(s) with von Willebrand factor-immunoreactive endothelial cells (Figure 1E) and were identified in close cell-to-cell contact with lymph node cells (LNCs), such as CD11c^+^ dendritic cells (DCs) (Figure 1F), F4/80^+^ professional phagocytes (Figure 1G) and major histocompatibility complex (MHC) class II^+^ immune cells (Figure 1H). While GFP^+^ NPCs and neighbouring LNCs maintained mutually exclusive fluorescence patterns (Figure 1F and H), only very few phagocytes (5–10% of the total GFP^+^ cells in lymph nodes) were double positive for the GFP and the phagocyte markers F4/80 or CD11b. This suggested that a very low number of s.c.-injected NPCs might have undergone phagocytosis (Figure 1G and Figure S2).

**Figure 2:**
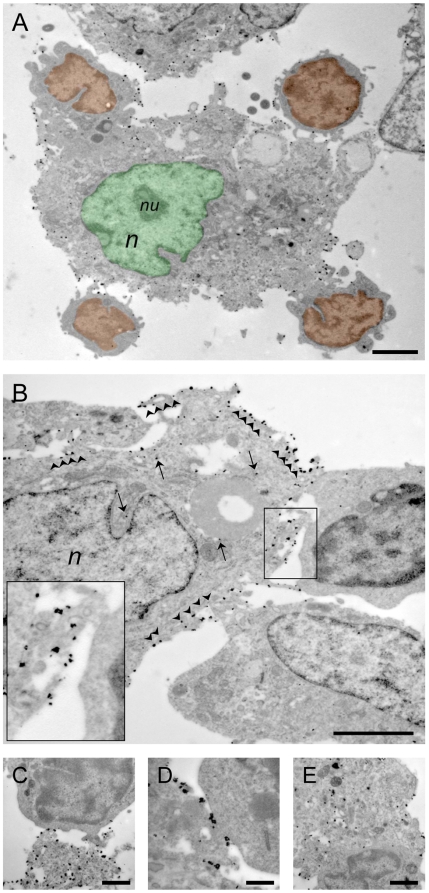
Electron microscopic in vivo appearance of s.c.-injected NPCs in draining lymph nodes. A, Transmission electron microscopy (TEM) images a large-size immunogold-labelled GFP^+^ NPC within a cervical lymph node of a R-EAE mouse at 72 days after cell injection. These GFP^+^ cells display morphological and ultrastructural features similar to individual NPCs from neurospheres in vitro (see also *Figure S3*). The NPC (pseudocolor green) takes contact with four individual lymph node cells (pseudocolor orange). The NPC shows an irregular and invaginated nucleus (*n*) with a single nucleolus (*nu*), and its cytoplasm contains abundant organelles; B, Individual GFP^+^ NPCs establishing anatomical contacts with two GFP^−^ lymph node cells–one of which (upper right) possesses the morphological characteristics of lymph blasts–through polarized microvilli (boxed area). Note the electrodense grain precipitates within the cytoplasm (arrows) and on the membrane (arrowheads and boxed area); C–E, NPCs in lymph nodes establish cell-to-cell contacts with lymph blasts through cytoplasmic expansions (C) or elongated intercellular junctions (D). Transplanted NPCs are occasionally found in deeper contact and enclosing resident lymph cells (E). Images refer to representative draining lymph nodes from R-EAE mice injected s.c. with NPCs at 3 and 10 dpi. Scale bars in A and B: 2 µm, in C and E: 1 µm, and in D: 500 nm.

The ultrastructural analysis of lymph nodes from NPC-injected R-EAE mice confirmed the presence of numerous large-size GFP^+^ cells with electrodense grain precipitates displaying morphological and ultrastructural features similar to neurospheres in vitro (Figure 2 and Figure S3). These GFP^+^ cell were rich in organelles, displayed an endoplasmic reticulum with short cisterns and invaginated nucleus with clumped chromatin and were frequently found in close contact with LNCs (Figure 2A). Individual GFP^+^ NPCs were found to establish consistent anatomical contacts with LNCs through either polarized microvilli (*nanotubes*) (Figure 2B), cytoplasmic expansions (Figure 2C) or elongated intercellular junctions (Figure 2D). Transplanted NPCs were also occasionally found in deeper contact and enclosing resident lymph cells, up to membrane fusion (Figure 2E), and infrequently, GFP^+^ NPCs showed cytoplasmic vacuoles and picnotic nuclei (Figure S2).

### Establishment of ectopic germinal niche-like areas in lymph nodes

We then sought to investigate the molecular features of the lymph node areas appearing as preferential sites of accumulation and, importantly, long term persistence of s.c.-injected NPCs.

**Figure 3:**
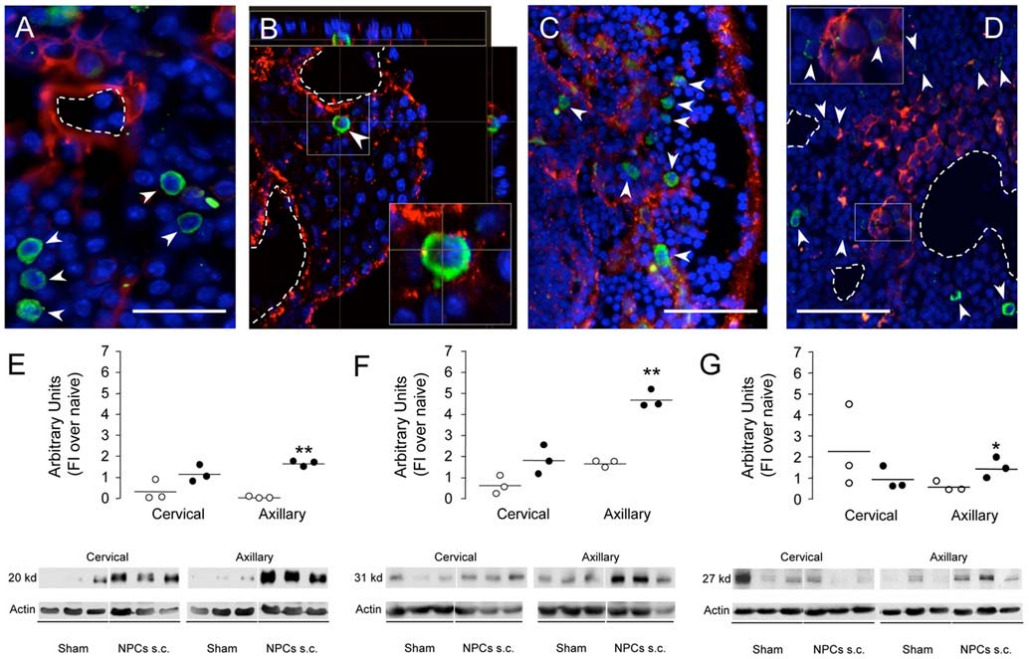
Establishment of atypical perivascular lymph node niches. A–D, Representative confocal microscope images of the persistence of s.c.-injected NPCs (arrowheads, green) within lymph node perivascular areas in R-EAE mice at 75 dpi. Focal expression of tenascin C (A, red), BMP-4 (B, red), BMP-7 (C, red) and Shh (D, red) are shown. Dashed lines represent blood vessels. Nuclei in A–D are counterstained with DAPI. Scale bars: A, 50 µm; C and D, 100 µm. E–G, Western blot analysis of BMP-4 (E), Noggin (F) and Shh (G) expression in cervical and axillary lymph nodes from sham-treated (white dots) and NPC-injected (black dots) R-EAE mice at 2 weeks after treatment. Results from individual mice (n = 3/group) are represented as dots and expressed as protein Arbitrary Units (AU, fold induction over average naive) (±SEM). Data refer to R-EAE mice injected s.c. with NPCs at 3 and 10 dpi and sacrificed at two weeks after cell injection. *p≤0.05; **p≤0.005, vs. sham-treated controls.

Interestingly, lymph nodes from R-EAE mice showed focal high (re)expression of extracellular matrix proteins typical of germinal CNS niches, such as tenascin C [14], as well as major stem cell regulators, such as BMP-4 and -7 (but not BMP-2) [15], [16], and Shh [17], [18] (Figure 3A–D), when compared to lymph nodes from control non-EAE mice. Among these regulators, only BMP-4 (Figure 3E), the BMP antagonist Noggin (Figure 3F), and Shh (Figure 3G) were significantly increased (by 3 to 4-fold) in the axilliary lymph nodes from mice s.c.-injected with NPCs, as compared to sham-treated controls.

We then hypothesised that the establishment of a permissive *ectopic germinal niche-like* micro-environment in perivascular lymph node areas–molecularly reminiscent of that described in the adult brain upon NPC transplantation [6], [19], [20] –might have allowed the survival, and possibly sustained the function, of s.c.-injected NPCs.

### Inhibition of the generation of encephalitogenic T cells

We next investigated whether the observed close vicinity (and interaction) between s.c.-injected NPCs and LNCs within favourable lymph node microenvironments might have had major immunological effects, such as the impairment of the generation of effector (CD4^+^) T cells responsible for the chronic CNS-confined inflammation observed in EAE [21].

**Figure 4:**
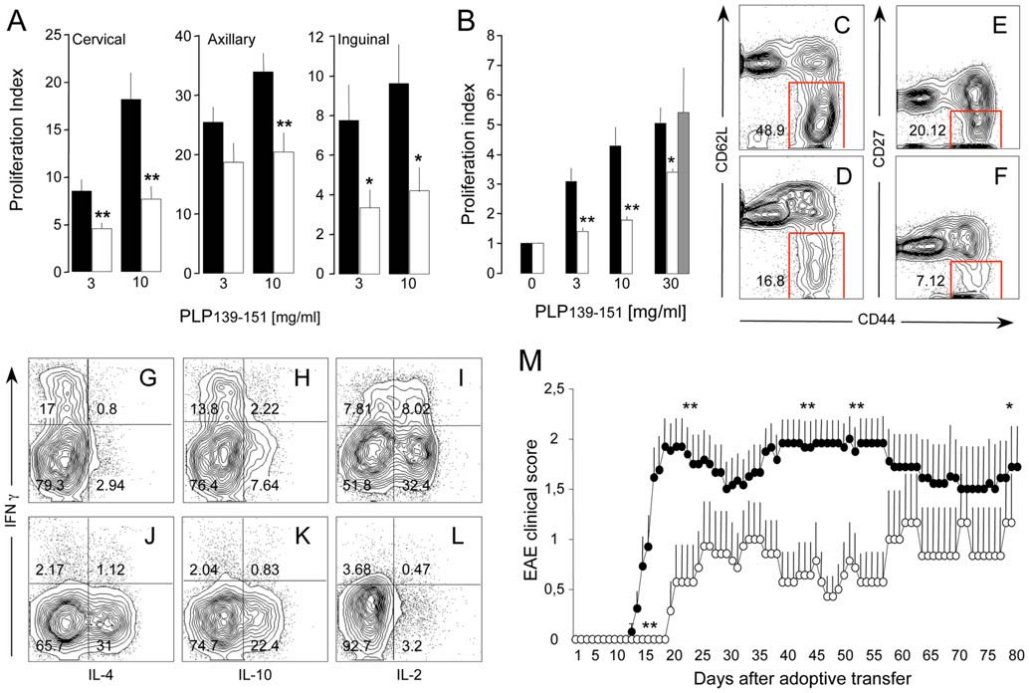
NPCs inhibit generation of effector T cells. A, Ex vivo proliferation of lymph node cells (LNCs) from sham- (black bars) and NPC-treated (white bars) R-EAE mice. Mice (n = 15 mice/group) were treated with either the carrier solution or NPC s.c. at 3 dpi and sacrificed at 10 dpi; B, Response to PLP139–151 of LNCs co-cultured with NPCs (LNCs/NPCs) either in the same well (white bars) or in a trans-well system (grey bars). Note the significant suppression of proliferation, compared to control LNCs (black bars). No significant interference with T cell response is observed in trans-well experiments. Data in A and B are mean Proliferation Index (over basal proliferation) (±SEM) from a total of n≥3 independent experiments. *p≤0.05; **p≤0.005, vs. control LNCs; C–F, LNCs/NPCs (D and F) show reduced percentages of CD44^high^/CD62L^−^ and CD44^high^/CD27^−^ CD4^+^ effector memory T cells, compared to control LNCs (C and E); G–L, LNCs/NPCs show reduced percentages of IFN-γ- (y axis) and IL-2-producing (x axis in L) CD4^+^ T cells, while higher percentages IL-4- (x axis in J), IL-10-producing (x axis in K) CD4^+^ T cells, compared to control LNCs (G, H and I, respectively); M, Adoptive transfer to naive SJL recipients of PLP139–151-specific LNCs having being either co-cultured (LNCs/NPCs, white circles, n = 8 mice) or not (LNCs, black circles, n = 13 mice) with NPCs. Note the significant impairment of EAE development (e.g., delay of the disease onset and reduction of the clinical score), when disease is adoptively transferred with LNCs/NPCs. Data are mean clinical score (±SEM). *p≤0.05; **p≤0.005.

We first found that lymph node T cells from NPC-transplanted R-EAE mice displayed a significantly lower proliferation rate ex vivo in response to PLP139–151, as compared to T cells from sham-treated R-EAE control mice. The same magnitude of unresponsiveness was observed in cervical, axillary and inguinal lymph nodes (p≤0.05) (Figure 4A). Consistently, LNCs isolated from SJL mice 10 days after PLP139–151 immunization and co-cultured in vitro with NPCs (LNCs/NPCs) showed: (i) a significant impairment of PLP139–151-specific T cell proliferation [p≤0.05, vs. (*non co-cultured*) control LNCs] (Figure 4B); (ii) a decreased number of effector memory (*antigen-specific*) CD4^+^ CD44^high^/CD62L^−^ and CD44^high^/CD27^−^ T cells [22], [23] (Figure 4C–F); and, (iii) an increase of CD4^+^ (*antigen-specific*) T cells releasing the anti-inflammatory cytokines IL-4 and IL-10 (Figure 4G–L).

Quite distinct from what is described for immune regulatory somatic mesenchymal stem cells (MSCs), our findings with NPCs were not paralleled by an increase of T cell apoptosis [24], [25] and/or generation of CD4^+^/Foxp3^+^/CD25^+^ regulatory T cells [26], [27] (*data not shown*). Finally, LNCs from co-cultures with NPCs (LNCs/NPCs) were capable of adoptively transferring a significant milder R-EAE compared to control LNCs, as indicated by clinical (Figure 4M) and neuropathological findings (number of CNS inflammatory infiltrates per mm^2^: adoptive EAE transfer control LNCs, 11.1±1.5; adoptive LNCs/NPCs, 2.6±0.6; p≤0.0001; % of demyelination/mm^2^ of spinal cord: adoptive control LNCs, 5.6±0.0; adoptive LNCs/NPCs, 0.87±0.2; p≤0.0001; % of axonal loss/mm^2^ of spinal cord: adoptive control LNCs, 5.6±0.7; adoptive LNCs/NPCs, 0.63±0.2; p≤0.0001).

### Restrainance of dendritic cell maturation trough soluble bone morphogenetic proteins

The in vivo and in vitro finding that the expansion of antigen-specific effector T cells is hampered in lymph nodes from s.c.-injected R-EAE mice prompted us to speculate a causal impairment of the antigen presentation capacities of professional antigen presenting cells (APC), such as DCs.

**Figure 5:**
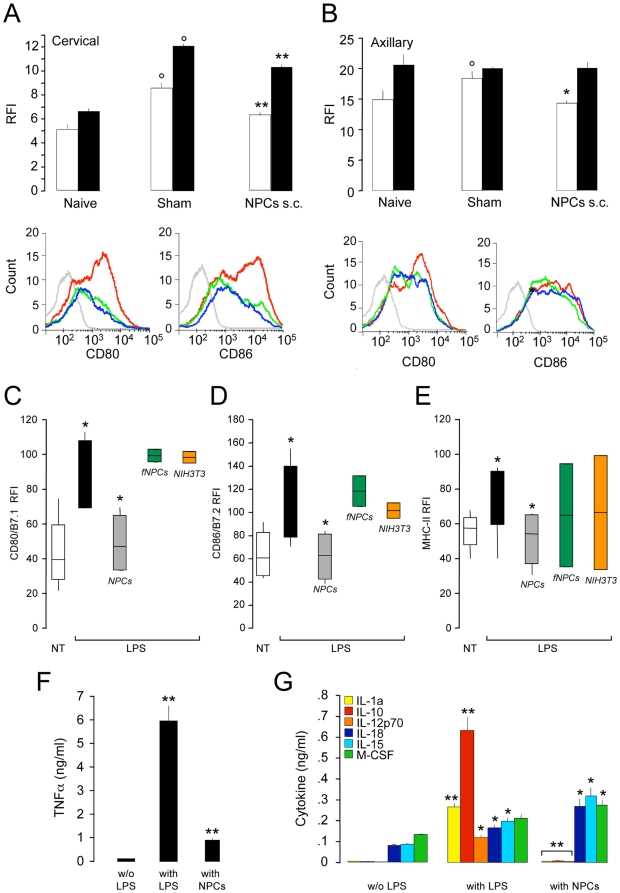
NPCs restrain DC maturation and cytokine production. A and B, Ex vivo FACS analysis of DCs from the lymph nodes of R-EAE mice treated with either the carrier solution or NPC s.c. at 3 dpi and sacrificed at 7 dpi. The injection of NPCs hinders the up-regulation of CD80 (white bars) and CD86 (black bars) on maturating DCs from cervical and axillary lymph nodes. Histograms in A and B are representative of the FACS analysis for CD80 and CD86 on CD11c^+^ lymph node DCs from cervical and axillary lymph nodes of naive (blue lines) and R-EAE mice either sham- (red lines) or NPC-treated (green lines). The grey lines are the fluorescence minus one (FMO) control staining. Data are mean relative fluorescence intensity (RFI) over FMO (±SEM) from a total of n = 2 independent experiments (n = 3 mice/group/experiment). °p≤0.05, vs. control DCs. *p≤0.05 and **p≤0.0005, vs. sham-treated controls; C–E, Live NPCs (grey bars), but not PFA-fixed NPCs (fNPCs, green bars) and live NIH3T3 mouse embryonic fibroblasts (orange bars), inhibit the up-regulation of CD80/B7.1 (C), CD86/B7.2 (D), and MHC-II (E) on DCs maturating in vitro with LPS. Cells were co-cultured with DCs (1∶1 NPC/DC ratio) in a trans-well system. White bars are not treated (NT) DCs, while black bars are control (*non co-cultured*) DCs. Data were obtained from a total of n≥3 independent experiments. Data in A–E are mean RFI over unstained (±SEM); F and G, Co-cultured NPCs significantly inhibit the release of TNFα (F), while completely suppressing the release of IL-1α, IL-12p70 and IL-10 (G), from DCs maturating in vitro with LPS. NPCs also favour the release of IL-15, IL-18 and M-CSF (G). Data are mean cytokine ng/ml (±SEM) from a total of n≥3 independent experiments. C–G, *p≤0.05; **p≤0.005, vs. control DCs.

Indeed, a significant down-regulation of the co-stimulatory molecules CD80/B7.1 and CD86/B7.2 (Figure 5A and B), but not MHC class-II, (*data not shown*) was found ex-vivo on DCs from NPC-treated R-EAE mice, as compared to DCs from sham-treated controls. This latter finding was further confirmed in vitro, as DCs maturating upon exposure to either tumor necrosis factor (TNF)-α or toll-like receptor (TLR) agonists [e.g., poly-IC, lipoteichoic acid (LTA) and bacterial lipopolysaccharide (LPS)] failed to up-regulate CD80/B7.1, CD86/B7.2 and MHC class-II (Figure 5C–E and Figure S4), while producing significantly lowers amounts of pro-inflammatory cytokines, when co-cultured with NPCs (Figure 5F–G and Figure S4). This effect was observed when DCs and NPCs were co-cultured both in the same dish as well as in a trans well co-culture system avoiding cell-to-cell contact (Figure 5C–G and Figures S4 and S5).

**Figure 6:**
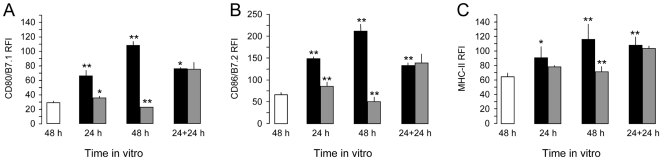
The NPC-mediated restrainance of DC maturation is reversible upon the establishment of appropriate maturation condition in vitro. The impairment of the expression of CD80/B7.1 (A), CD86/B7.2 (B) and MHC-II (C) by DCs maturating in vitro with LPS and being co-cultured with NPCs (DCs/NPCs, grey bars) is restored if NPCs are removed from the upper chamber after 24 hours and new medium with LPS is added for further 24 hours. White bars are not treated (NT) DCs, while black bars are control (*non co-cultured*) DCs. Data are mean RFI (±SEM) over unstained DCs from a total of n = 2 independent experiments. *p≤0.05; **p≤0.005, when either control DCs are compared to NT or DC/NPCs are compared to control DCs.

Interestingly enough, this impairment of the DC maturation was reversible and it could be fixed if the NPCs were removed from the co-culture in vitro system and DCs were put back on maturation with fresh LPS (Figure 6).

Therefore, it would be very likely that, at least part of the observed interference with DC maturation may be attributed to NPCs releasing soluble immune-like molecules.

Recent evidence has shown that major developmental factors controlling stem cell fate decisions during vertebrate embryogenesis and organogenesis–such as the Hedgehog (Hh) family proteins, and the BMPs-2 and -4–also play a role in regulating cell fate and determination in self-renewing tissues in adults, such as the immune system and the haematopoietic system. In one case, the involvement of the Hh signalling has been shown in the negative regulation of the T cell differentiation in the mouse thymus, both in the adult and the foetus [28], [29]. But the other hand, both Shh and BMP-2/-4 have been shown to regulate the development and proliferation of haematopoietic stem cells and the differentiation of mouse thymocytes [29], [30], [31].

Therefore, the finding that lymph node DCs from R-EAE mice are one of the preferential targets of the major environmental regulators being increased at the level of *ectopic germinal niche-like lymph node areas*, it is not completely surprising.

**Figure 7:**
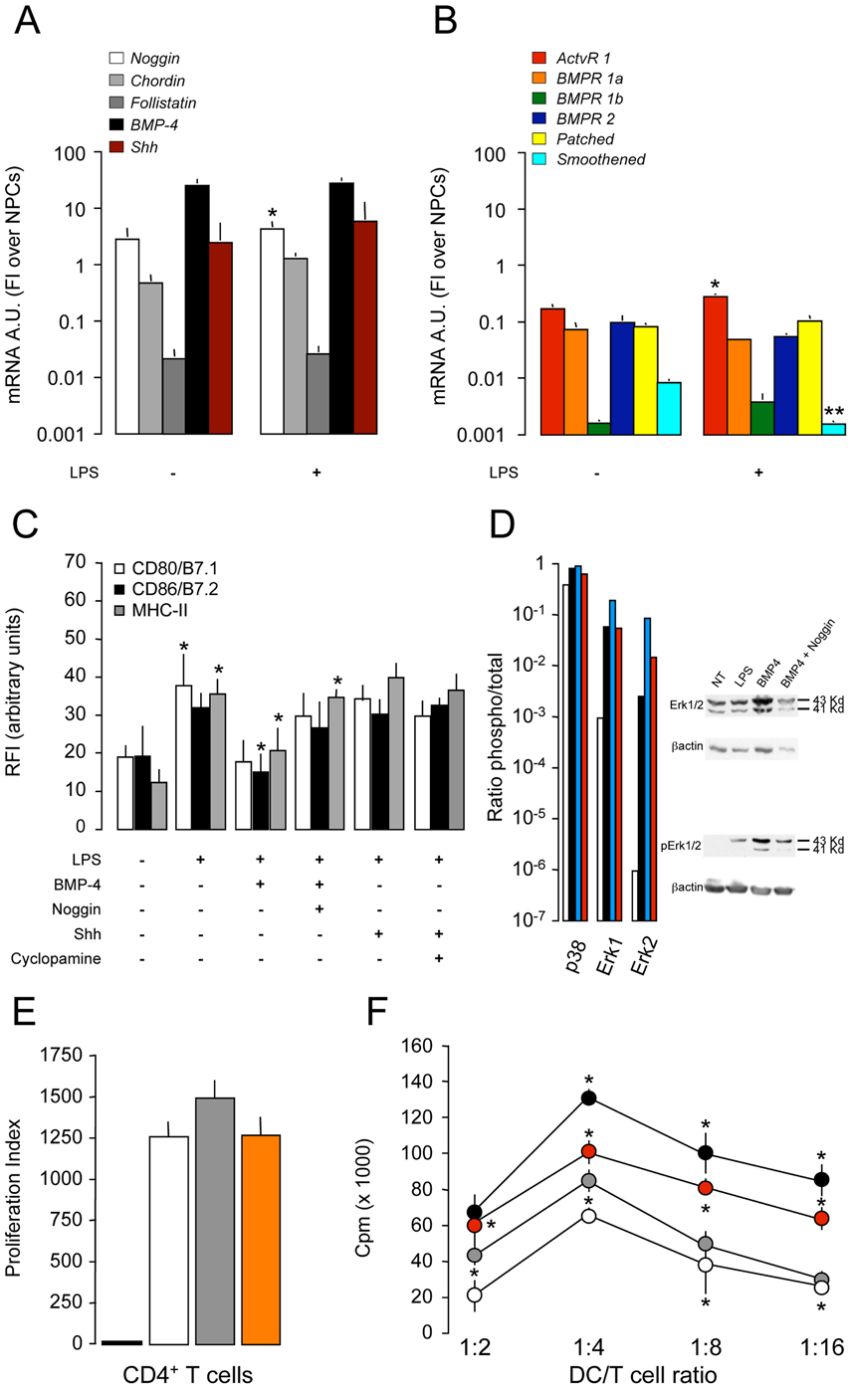
BMP-4-dependent hindrance of DC maturation. A and B, qRT-PCR analysis of BMPs/BMP antagonists, BMP receptors and Shh/Shh receptors in DCs. Note the significant up-regulation of mRNA levels for Noggin (A) and ActvR 1 (B) and down-regulation of the Shh receptor Smoothened (B) upon LPS activation of DCs. The mRNA levels of the other two BMP antagonists Chordin and Follistatin (A) remain unchanged after LPS. Data are mean mRNA arbitrary units (FI over NPCs) (±SEM) from a total of n≥3 independent experiments. *p≤0.05; **p≤0.005, vs. control DCs; C, Soluble recombinant BMP-4, but not recombinant Shh, inhibits the up-regulation of CD80/B7.1, CD86/B7.2, and MHC-II on DCs maturating with LPS. Almost complete reversion of the hindrance of DC maturation is obtained when soluble recombinant Noggin is added to the culture system. The addition of the Shh antagonist Cyclopamine did not exert any effect. Data are mean RFI (±SEM) from a total of n≥3 independent experiments. *p≤0.05, vs. control DCs; D, Immunoblot analysis of the TLR ligand-dependent activation of the MAPK p38 and Erk1/2 in DCs maturating with LPS in vitro. Increased phosphorilation of Erk1/2–and to a lower extent of p38^MAPK^–is obtained when BMP-4 (blue bars) is added to LPS-treated DCs (black bars). The effect is reverted by the addition of the BMP-4 antagonist Noggin (red bars). White bars are not treated (NT) DCs. Values are ratios of phosphorilated over total protein. Relative levels of protein expression were normalized to β actin; E, Proliferation to CD3/CD28 of lymph node CD4^+^ T cells from naive SJL mice in presence of BMP-4 (grey bar) or Shh (orange bar). Neither of the two morphogens interferes with CD4^+^ T cell response. The white bar is the proliferation to CD3/CD28 only, while the black bar is the basal proliferation (no CD3/CD28). Data are mean proliferation index (over basal proliferation) (±SEM) from a total of n = 3 independent experiments; F, Proliferation of CD4^+^ T cells from PLP139–151-immunized mice at first re-call with the antigen in vitro that are co-cultured with naïve DCs which have been pulsed with PLP139–151 during the last 18 hours of LPS-induced maturation (positive control, black circles). Significant reduction of antigen-specific proliferation of CD4^+^ T cells is observed when DCs are co-cultured with NPCs (white circles), while recovery of the antigen presenting efficiency is observed when the BMP-4 antagonist Noggin is added to the DC/NPC co-culture (red circles). Grey circles are T cells co-cultured with non-activated DCs. Data are mean counts per minute (cpm) (±SEM) from a total of n≥3 independent experiments. *p≤0.05, vs. positive control.

As such, DCs are found to express the whole apparatus (e.g., agonists, antagonists, receptors) for being both inducers as well as targets of both BMPs and Shh. Both machineries were also modulated at the mRNA level upon DC activation with LPS (Figure 7A and B).

Indeed, BMP-4, but not Shh, was capable of down modulating the membrane expression of CD80/B7.1, CD86/B7.2, and MHC class II molecule, when added in vitro to DCs with LPS (Figure 7C). Furthermore, the addition of the BMP antagonist Noggin to both not treated as well as DCs maturating with LPS did not exert any effect (Figure S6).

This BMP-4-dependent effect on DCs resulted in the activation of the mitogen-activated protein kinases (MAPK) p38 and Erk1/2, but not the classical Smad-dependent (*data not shown*) [32], intracellular signalling pathways and, again, it was blocked by the addition of Noggin (Figure 7D). As additional confirmation of the central role played by BMP-4 in DC function only, we found that Noggin (i) significantly reverted the inhibitory effect of BMP-4 on LPS-induced maturation of DCs (Figure 7C); and, (ii) restored both the expression of co-stimulatory molecules and the capacity to release pro-inflammatory cytokines of DCs co-cultured with NPCs (Figure S4). Moreover, neither BMP-4 nor Shh interfered with the proliferation of CD4^+^ T lymphocytes stimulated with CD3/CD28 in the absence of APCs (Figure 7E). As further functional proof, Noggin reverted the antigen presentation capacity of naive PLP-pulsed DCs co-cultured with NPCs in vitro (Figure 7F).

**Figure 8:**
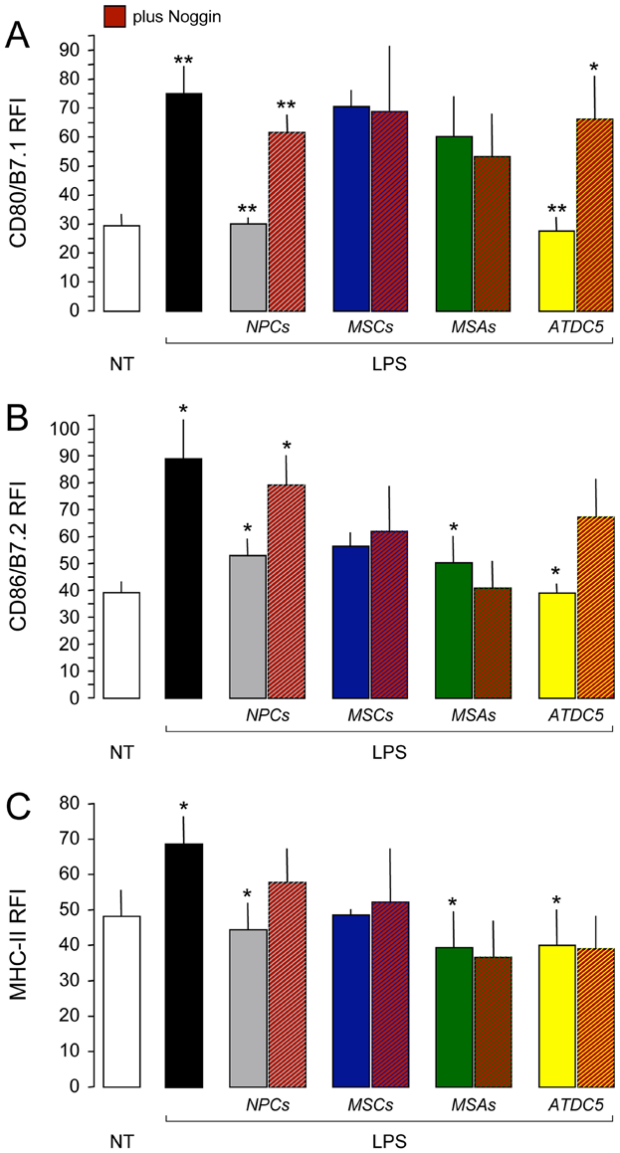
The BMP-4-dependent hindrance of DC maturation is specific for NPCs. A–C, NPCs (grey bars), but not bone marrow-derived MSCs (blue bars), or vessel associated MSAs (green bars), inhibit the up-regulation of CD80/B7.1 (A), CD86/B7.2 (B), and MHC-II (C) on DCs maturating in vitro with LPS. Almost complete recovery of the co-stimulatory molecule expression is obtained on DC/NPC only, when soluble recombinant Noggin is added to the co-culture system. Cells were co-cultured with DCs (1∶1 cell/DC ratio) in a trans-well system. White bars are not treated (NT) DCs, while black bars are control (*non co-cultured*) DCs. BMP-secreting ATDC5 condrogenic cells (orange bars) are used as positive control. Data were obtained from a total of n = 2 independent experiments. Data in are mean RFI over unstained (±SEM). *p≤0.05; **p≤0.005, when either control DCs are compared to NT, co-cultures are compared to control DCs or co-cultures plus Noggin are compared to co-cultures.

Then, in order to conclusively demonstrate whether the observed immune regulatory effect was indeed specific for NPCs, we challenged the capacity of other somatic stem cells, such as bone marrow derived MSCs and vessel-associated mesoangioblasts (MSAs), of interfering with the expression of co-stimulatory molecules, when co-cultured with DCs maturating in vitro with LPS. Interestingly enough, neither MSCs nor MSAs showed significant inhibitory capacity of the expression of CD80/B7.1 (Figure 8A), CD86/B7.2 (Figure 8B), and MHC-II (Figure 8C) on DCs, as compared to NPCs. More importantly, the addition of the BMP antagonist Noggin was capable to restore the high levels of co-stimulatory molecule expression when DCs were co-cultured with NPCs and the *BMP-secreting* [33] ATDC5 condrogenic precursor cells only (Figure 8A–C).

## Discussion

Somatic stem cell-based therapies–including those delivering NPCs–are broadly advised as potential alternative medicines aiming at, in this case brain repair for invalidating CNS diseases. This directive has arisen from the demonstration that NPC-driven clinico-pathological recovery is achieved in several pre-clinical models of neurological disorders [7]. While the replacement of lost/damaged cells was until few years ago assumed as the prime therapeutic mechanism of CNS stem cells [3], [13], it has now become clear that transplanted somatic stem cells may simultaneously instruct several therapeutic mechanisms amongst which cell replacement, itself, does not prevail. To understand and elucidate the overall therapeutic potential of stem cells in neurological diseases, namely the capacity of stem cells to adapt their fate and function(s) to specific different pathological conditions, we and others have recently proposed the concept of therapeutic plasticity [7].

The transplantation of different sources of somatic stem cells (e.g. NPCs [3], [4], [6], [13], haematopoietic stem cells (HSCs) [34], [35], mesenchymal stem cells (MSCs) [36], [37]), with very little (if any) capability of neural (trans) differentiation, has promoted diffuse CNS repair via intrinsic neuroprotective bystander capacities. This neuroprotective capability, mainly exerted by undifferentiated stem cells releasing in situ, a milieu of neuroprotective and immunomodulatory molecules, is temporally and spatially orchestrated by environmental needs. Moreover, more recent evidence, including the study herein, has suggested that the majority of the stem cell-mediated therapeutic effects in inflammatory CNS disorders are possibly taking place peripherally, at the level of immune relevant anatomical site, such as secondary lymphoid organs [8], [9], [36]. Yet, a current and comprehensive illustration of the different mechanism(s) by which such cells exert their therapeutic potential is lacking.

Herein we report the capacity of NPCs to target, and synergize with immune cells in draining lymph nodes, and have demonstrated this by utilizing a highly peculiar protocol of therapeutic passive (s.c.) NPC vaccination enhancing the capacity of injected NPCs to selectively target the major peripheral sites of immune surveillance (e.g., draining lymph nodes) in mice with EAE [38].

S.c.-injected NPCs protect mice from chronic CNS-confined autoimmunity via an immune regulatory mechanism that restrains the generation and expansion of pathogenic autoreactive T cells. Regardless of the timing and the number of injections administered, R-EAE mice injected with NPCs before the disease onset showed significant clinical improvement, as compared to sham-treated controls. By contrast, the delayed s.c. injection of NPCs did not produce any detectable clinical improvement, as compared to sham-treated controls. At this juncture, we do not have any clear-cut mechanistic explanation in support of the lack of ‘treatment effect’. However, based on broad evidence supporting the potential of somatic stem cell-based therapies for the treatment of neurological disorders [7], [39], is the notion that NPCs exert a broad regulatory capacity, that is dependent on the time of cell injection, and that may be differentially exerted to specifically interact with cells of different microenvironments in vivo [6], [8], [9], [36], [37], [40].

Further demonstrated is that the therapeutic potential is sustained in vivo by the accumulation, survival and long-term persistence exclusively at the level of secondary lymphoid organs of the transplanted NPCs, which, in turn, modulate the in situ increase of major stem cell fate determinants. In lymph nodes, the increased bioavailability of BMP-4, BMP-7, Shh, and the BMP antagonist Noggin, released by both transplanted NPCs and immune cells, promotes the survival and persistence of s.c.-injected NPCs outside the CNS, at the levels of *ectopic perivascular germinal niche-like* lymph node areas.

This latter is indeed a critical observation. It is in fact well established that somatic stem cells injected systemically in multifocal inflammatory CNS disorders, specifically accumulate and persist for months at the level of inflamed perivascular CNS areas, while promoting recovery [6], [41], [42], [43], [44], [45]. In these areas, named *CNS atypical ectopic niches*, a molecular cross talk between the transplanted stem cells and CNS cells (e.g., reactive astrocytes, inflamed endothelial cells and blood-borne infiltrating T cells) takes place. The great majority of transplanted stem cells survive within the *CNS atypical ectopic niches*, while displaying undifferentiated features, owing to the focal release of developmental stem cell regulators by immune cells and CNS resident cells [6]. Also, surviving stem cells promote tissue protection by releasing immunomodulatory and neurotrophic molecules [7]. Therefore, the establishment of *atypical ectopic niches* upon stem cell therapy, at either the level of the CNS or in peripheral immunologically relevant sites, might be realistically advised as the functional (pre) requisite for the long-term therapeutic activity of transplanted stem cells.

Furthermore, the observed excess of BMP-4 in lymph nodes significantly impaired the maturation and antigen presentation capacities of lymph node DCs, which, in turn, failed to sustain the expansion and full activation of effector (encephalitogenic) T cells. This effect was highly specific for BMP-4, as the BMP antagonist Noggin significantly reverted the inhibitory effect of BMP-4 alone. Interestingly enough, this BMP-4-dependent immune regulatory effect on DCs was also highly specific for NPCs–but not for MSCs or MSAs–as well as completely reversible after the removal of NPCs from the co-cultures.

Our results–along with the recent demonstration that intravenously-injected NPCs suppress T cell proliferation within secondary lymphoid organs [8] –provide additional mechanistic evidence that CNS stem cells can be manipulated to act as immune regulators in promoting CNS repair/protection. This is indeed the first identification of a member of the transforming growth factor (TGF)β/BMP family of stem cell developmental regulators as a novel tolerogenic factor released by immune regulatory NPCs. The phenomenon herein described may be indeed further exploited as an efficient tool for vaccination therapy in autoimmune inflammatory conditions, which result in widespread CNS damage.

Importantly though, our observation follows some very recent reports, which have started identifying some of the first molecular mechanisms responsible for the somatic stem cell-mediated regulatory effect on DCs. These have included the demonstration that MSCs affect the DCs function trough the release of (i) IL-6 inhibiting the differentiation to immature DCs [46], (ii) Notch ligand Jagged-2, which induces the generation of regulatory DCs [47] and, more recently, (iii) prostaglandin E2 inhibiting the differentiation to immature DCs [48]. Therefore, while providing a much deeper molecular view of the therapeutic plasticity of somatic stem cells, these findings and our own data also predict a fairly transversal (inter-disease) significance for most of the somatic stem cell-based approaches aiming at immune regulation.

Critically important is for promoting CNS repair/protection the ongoing study and the elucidation of the role(s) of CNS stem cells in regenerative medicine and it will contribute to the development of more efficacious (stem) cell-based therapies.

## Materials and Methods

### NPC derivation and cultures

Adult neurospheres were generated from the SVZ of four-to-eight week-old SJL mice, as described [6]. Further information is provided as Material S1.

### EAE induction and NPC transplantation

Relapsing EAE was induced in a total of n = 112 four-to-eight week-old (weight 20–25 grams) female SJL mice, as described [6]. At time of cell transplantation, single cell-dissociated NPCs were injected sub-cutaneously in both flanks (0.5×10^6^ cell/150 µl PBS in each site). Cell transplants with live NPCs were performed prior to disease onset–at 3 and 10 dpi (n = 35) or 10 dpi only (n = 22) –or after the disease onset, at 30 dpi (n = 10). Paraformaldehyde (PFA)-fixed [5% PFA for 5 minutes at room temperature (r.t.)] NPCs injected s.c. at 10 dpi (n = 10) were use as cellular controls. Sham-treated mice (n = 35) received 0.1 M PBS s.c. (150 µl in each site) at 3 and 10 dpi. All procedures involving animals were performed according to the guidelines of the Animal Ethical Committee of our Institute (IACUC no. 265 to S.P.).Further information is provided as Material S1.

### In vitro expansion and adoptive transfer of PLP139–151-specific mouse T cells

PLP139–151-specific T cells were obtained from the draining lymph nodes of PLP139–151-immunized SJL mice, at 10 dpi, as described [6]. Further information is provided as Material S1.

### Lymph node cell (LNC)/NPC co-cultures

LNCs were isolated from inguinal, axillary, and paraorthic lymph nodes of either naïve or PLP139–151-immunized mice at 10 dpi and suspended in RPMI complete medium. To examine the effects of NPCs at the time of antigen presentation, LNCs (7×10^5^/well) from PLP139–151-immunized mice were cultured in triplicate in 200 µl in 96 well flat bottom microtiter plates (Costar), stimulated with various concentrations of PLP139–151 (1–30 µg/ml) and incubated at 37°C for 4 days. In co-cultures, single cell-dissociated NPCs were added at 1∶2 NPC/LNC ratio either to the same well or to the upper chamber of a 0.4 µm membrane-separated trans-well system (Nunc). Proliferation of LNCs was determined by adding 1 µCi ^3^H-thymidine during the final 18 hours of the culture, as described. Data were expressed as mean proliferation index (over basal proliferation) (±SEM) from a total of n≥4 independent experiments.

### Dendritic cell (DC) preparation and co-cultures

Bone marrow-derived DCs were prepared from flushed tibias and femurs of naive SJL mice (Charles River) and propagated in vitro for 1 week in Iscove's medium (Invitrogen Life Technologies) supplemented with 100 U/ml penicillin (BioWhittaker), 100 µg/ml streptomycin (BioWhittaker), 1.5 mM L-glutamine (BioWhittaker), 10% heat-inactivated foetal calf serum (FCS; EuroClone) and recombinant mouse GM-CSF and IL-4 (both 25 µg/ml, R&D System), as described [49]. In co-cultures, to verify the effect of NPCs on DC maturation, single cell-dissociated NPCs were added (1∶1 NPC/DC ratio) at day 5, either in the same well or in the upper chamber of a 0.4 µm membrane-separated trans-well system (Nunc). Further information is provided as Material S1. PFA-fixed mouse NPCs, NIH3T3 embryonic fibroblasts [50], mouse mesoangioblasts (MSAs) [51], bone marrow-derived mouse mesenchymal stem cells (MSCs) [37] and the ATDC5 condrogenic cell line [52] (all 1∶1 cell/DC ratio) were used as cellular controls. Further information on MSA, MSC and ATDC5 cell preparations, culture and phenotype is provided as Material S1.

### Ex vivo characterization of DC cells from draining lymph nodes

Mice were sacrificed at 7 days post immunization and axillary, cervical and inguinal lymph nodes single-cell suspensions were prepared by pressing the tissue through nylon cell strainer (70 µm, BD Falcon). Cell suspensions were washed with complete media and counted. To analyze CD11c^+^ DC population the following antibodies were used: CD11c (clone HL3), CD86 (clone GL1), CD80 (clone 16-10A1), or MHC class II (clone M5/114.15.2). All antibodies were conjugated to PE or APC and were purchased from BD Biosciences/Pharmingen or Cedarlaine. For the analysis of cell surface protein expression, 1,5×10^6^ lymph node cells were stained with the antibodies above for 15 min at room temperature in presence of 2 µg/ml of mouse IgG as FcR blocking reagent. Cells were then washed in PBS and just before analysis on a FACSCanto TM (Becton Dickinson). 7-Amino-actinomycin D (7-AAD) was added for dead cell exclusion. A maximum of 5,000 events into the gate of CD11c cells were acquired for each sample. Data were analyzed using FCS Express V3 (De Novo Software).

### RT-PCR

Real-time quantitative PCR was performed using pre-developed Taqman™ Assay Reagents on an ABI Prism™ 7700 Sequence Detection System (Applied Biosystems) according to manufacturers protocol. Further information is provided as Material S1.

### Western blot analysis

Western blot analysis of BMP-4, Noggin and Shh was performed using surgically dissected pairs of cervical and axillary lymph nodes from naive SJL mice and both sham- and NPC-treated R-EAE mice. Analysis of ERK1/2, phospho ERK1/2, p38, phospho p38, SMAD 1 and phospho-SMAD1/5/8 proteins expression was performed using not treated and LPS-treated DCs. Further information is provided as Material S1.

### Cytokine assay

Supernatants from DC/NPC co-colture experiments were collected and cytokine production was analyzed with the Bioplex Protein Array system (BioRad) using fluorescent beads specific for mouse IL-1α, IL-2, IL-4, IL-10, IL-12 (p70), IFN-γ, TNF-α, IL-15, IL-18 and M-CSF, according to the manufacturer instructions as described [53]. Samples were analysed in quadruplicate, and fluorescence was read with the Luminex system (Biorad). Bio-plex manager 4.0 software (Biorad) was used for the analysis of data.

### FACS analysis

FACS analyses were carried out on a BD FACSCanto™ (Becton Dickinson), a FACSCalibur (Becton Dickinson) and a CyAn™ ADP (Dako), and data were analyzed using FlowJo (Treestar) and CellQuest (BD Biosciences) software. At least 30.000 events were acquired for each sample. Complete list of antibodies and further information are provided as Material S1.

### Tissue pathology

At sacrifice mice were trans-cardially perfused with 4% paraformaldehyde and CNS tissue (brain and spinal cord), liver, spleen, kidneys and cervical, inguinal, axillary, mesenteric and paraortic lymph nodes were removed and processed for pathology on either paraffin-embedded or frozen tissue samples, as described [6]. The complete list of antibodies used and further information is provided as Material S1.

### Immunogold and electron microscopy

At sacrifice, mice were perfused transcardially with 0.9% saline, followed by 4% paraformaldehyde. The lymph nodes were removed and post-fixed in the same fixative overnight. Further information is provided as Material S1.

### Histochemistry

Further information is provided as Material S1.

### Statistical analysis

Data were compared using the Student's t-test for paired or unpaired data, the one-way Anova test or the Mann-Whitney U-test for non-parametric data.

## Supporting Information

Figure S1 A, EAE clinical score of PLP139-151-immunized SJL mice, either sham-treated (black circles) or transplanted s.c. with different NPC types. Only mice receiving passive vaccination with live NPCs at both 3 and 10 dpi (white circles) and 10 dpi only (grey circles) show pronounced clinical amelioration, when compared to sham-treated controls. Delayed (namely 30 dpi, red circles) s.c. live NPCs or paraformaldehyde-fixed s.c. NPCs at 10 dpi (blue circles) did not produce any detectable clinical improvement. Data are mean clinical score (±SD) from a total of n = 2 independent experiments. B, Clinical features of R-EAE mice injected s.c. with different NPC types. Data are mean numbers (±SEM) from a total of n = 2 independent experiments *p<0.05; **p<0.005, vs. sham-treated controls.
(2.65 MB TIF)

Figure S2 Phenotypical and morphological analysis of s.c.-injected NPCs accumulating into draining lymph nodes of R-EAE mice. A, Representative image of three s.c.-injected GFP+NPCs (arrowheads) co-expressing doublecortin (DCX) within a cervical lymph node. Scale bar: 40 µm. B, Representative image of two distinct lymph node CD11b+professional phagocytes being immune reactive also for GFP+. Scale bar: 10 µm. C, Transmission electron microscopy (TEM) of a vacuolized picnotic GFP+cell in a representative axillary lymph node. Note the presence of electron dense granules both in cytoplasm and cell surface. Scale bar: 2 µm. Images in A–C refer to representative draining lymph nodes from R-EAE mice injected s.c. with NPCs at 3 and 10 dpi and sacrificed at 72 days after cell injection.
(2.07 MB TIF)

Figure S3 TEM image of a NPC from a neurosphere in vitro. Note the irregular nucleus, and the organelle-rich cytoplasm with abundant mitochondria and endoplasmic reticulum. Morphological and ultrastructural features of this single NPCs in vitro are similar to the NPCs detected in vivo in lymph nodes (see also Figure 2). Scale bar: 2 µm.
(4.74 MB TIF)

Figure S4 The BMP antagonist Noggin reverts the hindrance of DC maturation and cytokine production. A–C, Noggin (red bars) almost completely reverts the down-regulation of CD80/B7.1 (A), CD86/B7.2 (B), and MHC-II (C) obtained when DCs maturating in vitro with different TLR agonists are co-cultured in trans-wells with NPCs (white bars) (see also Figure 3). Black bars are non co-cultured control DCs, while NT are not treated DCs. Data are expressed as mean RFI over unstained (±SEM) from n>4 independent experiments. D, The addition of Noggin induces substantial recovery of cytokine levels, whose production is impaired in DC/NPC co-cultures. Data are mean cytokine levels (ng/ml) (±SEM) from a total of n>3 independent experiments. *p<0.05 and **p<0.005, vs. control DCs.
(2.19 MB TIF)

Figure S5 NPCs inhibit the up-regulation of co-stimulatory molecules upon LPS activation of DCs. Representative histograms showing the fluorescence intensity for CD80/B7.1 (red lines), CD86/B7.2 (green lines) and MHC-II (blue lines) on untreated (NT) DCs, DCs activated with LPS and DCs activated with LPS and co-cultured with NPCs in the same well. Black lines represent isotype controls.
(8.69 MB TIF)

Figure S6 The BMP antagonist Noggin alone does not interfere with DC maturation in vitro. Soluble recombinant Noggin does not interfere with the expression of CD80/B7.1 and CD86/B7.2 onto DCs undergoing maturation with LPS in vitro. Data are mean RFI (±SEM) from a total of n = 2 independent experiments. **p<0.005, vs. control DCs.
(1.45 MB TIF)

Figure S7 Phenotype of NPCs (A), bone marrow-derived MSCs (B), vessel-associated MSAs (C) and ATDC5 condrogenic cells (D). Histograms demonstrating the expression of surface molecules (red) are overlaid with unstained controls (black).
(2.77 MB TIF)

Material S1 Supplementary Materials and Methods
(0.08 MB DOC)

## References

[1] Franklin RJ, Ffrench-Constant C (2008). Remyelination in the CNS: from biology to therapy.. Nat Rev Neurosci.

[2] Ben-Hur T, Goldman SA (2008). Prospects of cell therapy for disorders of myelin.. Ann N Y Acad Sci.

[3] Pluchino S, Quattrini A, Brambilla E, Gritti A, Salani G (2003). Injection of adult neurospheres induces recovery in a chronic model of multiple sclerosis.. Nature.

[4] Einstein O, Karussis D, Grigoriadis N, Mizrachi-Kol R, Reinhartz E (2003). Intraventricular transplantation of neural precursor cell spheres attenuates acute experimental allergic encephalomyelitis.. Mol Cell Neurosci.

[5] Aharonowiz M, Einstein O, Fainstein N, Lassmann H, Reubinoff B (2008). Neuroprotective effect of transplanted human embryonic stem cell-derived neural precursors in an animal model of multiple sclerosis.. PLoS ONE.

[6] Pluchino S, Zanotti L, Rossi B, Brambilla E, Ottoboni L (2005). Neurosphere-derived multipotent precursors promote neuroprotection by an immunomodulatory mechanism.. Nature.

[7] Martino G, Pluchino S (2006). The therapeutic potential of neural stem cells.. Nat Rev Neurosci.

[8] Einstein O, Fainstein N, Vaknin I, Mizrachi-Kol R, Reihartz E (2007). Neural precursors attenuate autoimmune encephalomyelitis by peripheral immunosuppression.. Ann Neurol.

[9] Lee ST, Chu K, Jung KH, Kim SJ, Kim DH (2008). Anti-inflammatory mechanism of intravascular neural stem cell transplantation in haemorrhagic stroke.. Brain.

[10] Fainstein N, Vaknin I, Einstein O, Zisman P, Ben Sasson SZ (2008). Neural precursor cells inhibit multiple inflammatory signals.. Mol Cell Neurosci.

[11] Ren G, Zhang L, Zhao X, Xu G, Zhang Y (2008). Mesenchymal stem cell-mediated immunosuppression occurs via concerted action of chemokines and nitric oxide.. Cell Stem Cell.

[12] Block GJ, Ohkouchi S, Fung F, Frenkel J, Gregory C (2008). Multipotent Stromal Cells (MSCs) are Activated to Reduce Apoptosis in Part by Upregulation and Secretion of Stanniocalcin-1 (STC-1).. Stem Cells.

[13] Ben-Hur T, Einstein O, Mizrachi-Kol R, Ben-Menachem O, Reinhartz E (2003). Transplanted multipotential neural precursor cells migrate into the inflamed white matter in response to experimental autoimmune encephalomyelitis.. Glia.

[14] Garcion E, Faissner A, ffrench-Constant C (2001). Knockout mice reveal a contribution of the extracellular matrix molecule tenascin-C to neural precursor proliferation and migration.. Development.

[15] Lim DA, Tramontin AD, Trevejo JM, Herrera DG, Garcia-Verdugo JM (2000). Noggin antagonizes BMP signaling to create a niche for adult neurogenesis.. Neuron.

[16] Colak D, Mori T, Brill MS, Pfeifer A, Falk S (2008). Adult neurogenesis requires Smad4-mediated bone morphogenic protein signaling in stem cells.. J Neurosci.

[17] Lai K, Kaspar BK, Gage FH, Schaffer DV (2003). Sonic hedgehog regulates adult neural progenitor proliferation in vitro and in vivo.. Nat Neurosci.

[18] Machold R, Hayashi S, Rutlin M, Muzumdar MD, Nery S (2003). Sonic hedgehog is required for progenitor cell maintenance in telencephalic stem cell niches.. Neuron.

[19] Lois C, Garcia-Verdugo JM, Alvarez-Buylla A (1996). Chain migration of neuronal precursors.. Science.

[20] Wichterle H, Garcia-Verdugo JM, Alvarez-Buylla A (1997). Direct evidence for homotypic, glia-independent neuronal migration.. Neuron.

[21] Zeine R, Heath D, Owens T (1993). Enhanced response to antigen within lymph nodes of SJL/J mice that were protected against experimental allergic encephalomyelitis by T cell vaccination.. J Neuroimmunol.

[22] Pope JG, Karpus WJ, VanderLugt C, Miller SD (1996). Flow cytometric and functional analyses of central nervous system-infiltrating cells in SJL/J mice with Theiler's virus-induced demyelinating disease. Evidence for a CD4+ T cell-mediated pathology.. J Immunol.

[23] Zeine R, Owens T (1992). Direct demonstration of the infiltration of murine central nervous system by Pgp-1/CD44high CD45RB(low) CD4+ T cells that induce experimental allergic encephalomyelitis.. J Neuroimmunol.

[24] Augello A, Tasso R, Negrini SM, Amateis A, Indiveri F (2005). Bone marrow mesenchymal progenitor cells inhibit lymphocyte proliferation by activation of the programmed death 1 pathway.. Eur J Immunol.

[25] Plumas J, Chaperot L, Richard MJ, Molens JP, Bensa JC (2005). Mesenchymal stem cells induce apoptosis of activated T cells.. Leukemia.

[26] Prevosto C, Zancolli M, Canevali P, Zocchi MR, Poggi A (2007). Generation of CD4+ or CD8+ regulatory T cells upon mesenchymal stem cell-lymphocyte interaction.. Haematologica.

[27] Maccario R, Podesta M, Moretta A, Cometa A, Comoli P (2005). Interaction of human mesenchymal stem cells with cells involved in alloantigen-specific immune response favors the differentiation of CD4+ T-cell subsets expressing a regulatory/suppressive phenotype.. Haematologica.

[28] Outram SV, Varas A, Pepicelli CV, Crompton T (2000). Hedgehog signaling regulates differentiation from double-negative to double-positive thymocyte.. Immunity.

[29] Varas A, Hager-Theodorides AL, Sacedon R, Vicente A, Zapata AG (2003). The role of morphogens in T-cell development.. Trends Immunol.

[30] Hager-Theodorides AL, Outram SV, Shah DK, Sacedon R, Shrimpton RE (2002). Bone morphogenetic protein 2/4 signaling regulates early thymocyte differentiation.. J Immunol.

[31] Bhardwaj G, Murdoch B, Wu D, Baker DP, Williams KP (2001). Sonic hedgehog induces the proliferation of primitive human hematopoietic cells via BMP regulation.. Nat Immunol.

[32] Zhou Q, Heinke J, Vargas A, Winnik S, Krauss T (2007). ERK signaling is a central regulator for BMP-4 dependent capillary sprouting.. Cardiovasc Res.

[33] Shukunami C, Akiyama H, Nakamura T, Hiraki Y (2000). Requirement of autocrine signaling by bone morphogenetic protein-4 for chondrogenic differentiation of ATDC5 cells.. FEBS Lett.

[34] Herrmann MM, Gaertner S, Stadelmann C, van den Brandt J, Boscke R (2005). Tolerance induction by bone marrow transplantation in a multiple sclerosis model.. Blood.

[35] Zhang J, Li Y, Chen J, Cui Y, Lu M (2005). Human bone marrow stromal cell treatment improves neurological functional recovery in EAE mice.. Exp Neurol.

[36] Gerdoni E, Gallo B, Casazza S, Musio S, Bonanni I (2007). Mesenchymal stem cells effectively modulate pathogenic immune response in experimental autoimmune encephalomyelitis.. Ann Neurol.

[37] Zappia E, Casazza S, Pedemonte E, Benvenuto F, Bonanni I (2005). Mesenchymal stem cells ameliorate experimental autoimmune encephalomyelitis inducing T-cell anergy.. Blood.

[38] Weller RO, Engelhardt B, Phillips MJ (1996). Lymphocyte targeting of the central nervous system: a review of afferent and efferent CNS-immune pathways.. Brain Pathol.

[39] Uccelli A, Moretta L, Pistoia V (2008). Mesenchymal stem cells in health and disease.. Nat Rev Immunol.

[40] Einstein O, Grigoriadis N, Mizrachi-Kol R, Reinhartz E, Polyzoidou E (2006). Transplanted neural precursor cells reduce brain inflammation to attenuate chronic experimental autoimmune encephalomyelitis.. Exp Neurol.

[41] Garbuzova-Davis S, Willing AE, Zigova T, Saporta S, Justen EB (2003). Intravenous administration of human umbilical cord blood cells in a mouse model of amyotrophic lateral sclerosis: distribution, migration, and differentiation.. J Hematother Stem Cell Res.

[42] Chu K, Kim M, Park KI, Jeong SW, Park HK (2004). Human neural stem cells improve sensorimotor deficits in the adult rat brain with experimental focal ischemia.. Brain Res.

[43] Xiao J, Nan Z, Motooka Y, Low WC (2005). Transplantation of a novel cell line population of umbilical cord blood stem cells ameliorates neurological deficits associated with ischemic brain injury.. Stem Cells Dev.

[44] Liu H, Honmou O, Harada K, Nakamura K, Houkin K (2006). Neuroprotection by PlGF gene-modified human mesenchymal stem cells after cerebral ischaemia.. Brain.

[45] Ziv Y, Avidan H, Pluchino S, Martino G, Schwartz M (2006). Synergy between immune cells and adult neural stem/progenitor cells promotes functional recovery from spinal cord injury.. Proc Natl Acad Sci U S A.

[46] Djouad F, Charbonnier LM, Bouffi C, Louis-Plence P, Bony C (2007). Mesenchymal stem cells inhibit the differentiation of dendritic cells through an interleukin-6-dependent mechanism.. Stem Cells.

[47] Zhang B, Liu R, Shi D, Liu X, Chen Y (2009). Mesenchymal stem cells induce mature dendritic cells into a novel Jagged-2-dependent regulatory dendritic cell population.. Blood.

[48] Spaggiari GM, Abdelrazik H, Becchetti F, Moretta L (2009). Mesenchymal stem cells (MSC) inhibit monocyte-derived dendritic cell (DC) maturation and function by selectively interfering with the generation of immature DCs: central role of MSC-derived prostaglandin E2.. Blood.

[49] Rovere-Querini P, Capobianco A, Scaffidi P, Valentinis B, Catalanotti F (2004). HMGB1 is an endogenous immune adjuvant released by necrotic cells.. EMBO Rep.

[50] Valentinis B, Capobianco A, Esposito F, Bianchi A, Rovere-Querini P (2008). Human recombinant heat shock protein 70 affects the maturation pathways of dendritic cells in vitro and has an in vivo adjuvant activity.. J Leukoc Biol.

[51] Sampaolesi M, Torrente Y, Innocenzi A, Tonlorenzi R, D'Antona G (2003). Cell therapy of alpha-sarcoglycan null dystrophic mice through intra-arterial delivery of mesoangioblasts.. Science.

[52] Horiguchi M, Akiyama H, Ito H, Shigeno C, Nakamura T (2000). Tumour necrosis factor-alpha up-regulates the expression of BMP-4 mRNA but inhibits chondrogenesis in mouse clonal chondrogenic EC cells, ATDC5.. Cytokine.

[53] Kerr JR, Cunniffe VS, Kelleher P, Coats AJ, Mattey DL (2004). Circulating cytokines and chemokines in acute symptomatic parvovirus B19 infection: negative association between levels of pro-inflammatory cytokines and development of B19-associated arthritis.. J Med Virol.

