# Supplementary figures and images for: Immune Regulatory Neural Stem/Precursor Cells Protect from Central Nervous System Autoimmunity by Restraining Dendritic Cell Function
Source: PLoS One. 2009 Jun 19;4(6):e5959. doi: 10.1371/journal.pone.0005959 (PMC2694997; doi:10.1371/journal.pone.0005959)

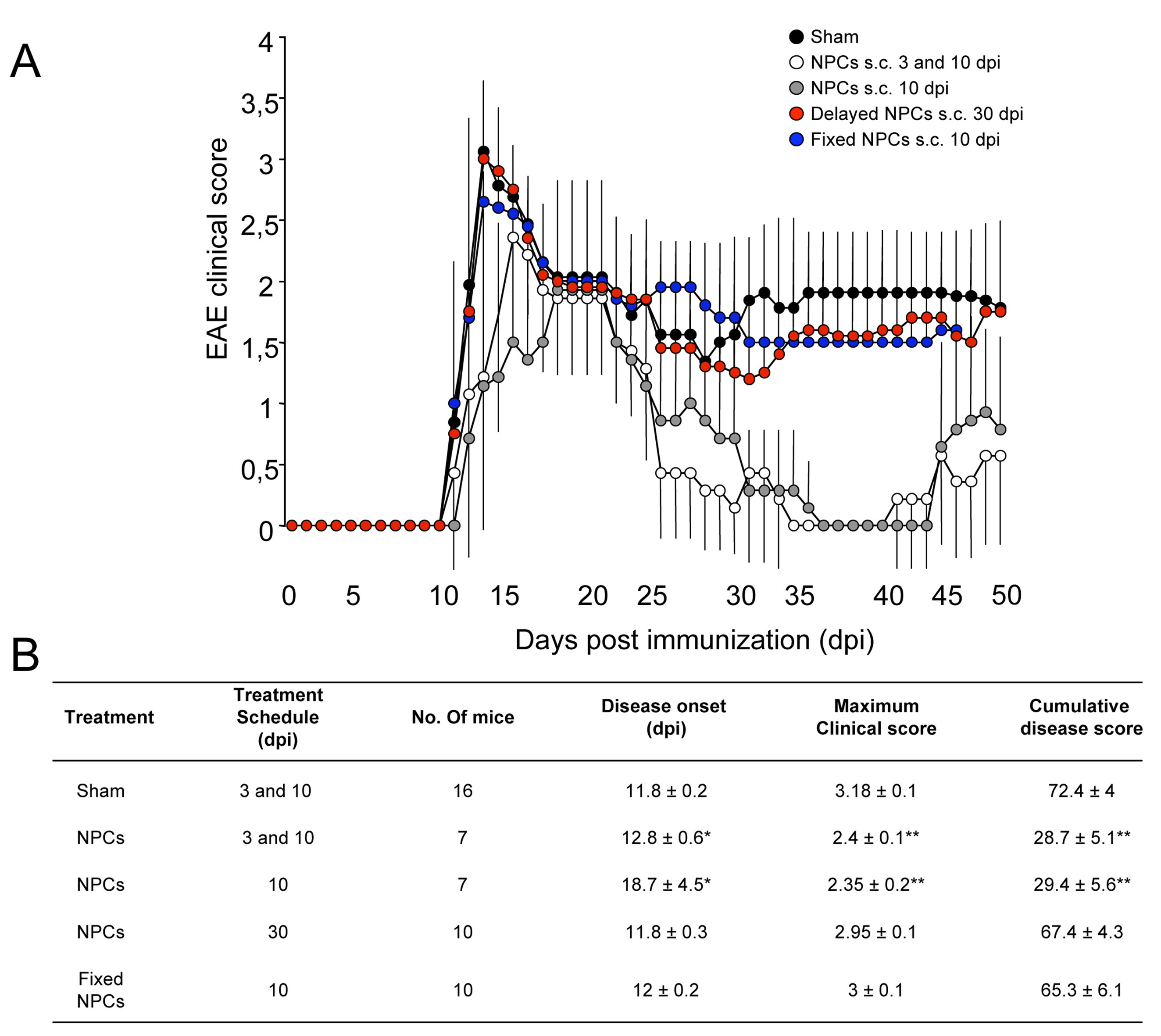

Supplement: Figure S1 — A, EAE clinical score of PLP139-151-immunized SJL mice, either sham-treated (black circles) or transplanted s.c. with different NPC types. Only mice receiving passive vaccination with live NPCs at both 3 and 10 dpi (white circles) and 10 dpi only (grey circles) show pronounced clinical amelioration, when compared to sham-treated controls. Delayed (namely 30 dpi, red circles) s.c. live NPCs or paraformaldehyde-fixed s.c. NPCs at 10 dpi (blue circles) did not produce any detectable clinical improvement. Data are mean clinical score (±SD) from a total of n = 2 independent experiments. B, Clinical features of R-EAE mice injected s.c. with different NPC types. Data are mean numbers (±SEM) from a total of n = 2 independent experiments *p<0.05; **p<0.005, vs. sham-treated controls. (2.65 MB TIF) [file pone.0005959.s001.tif]

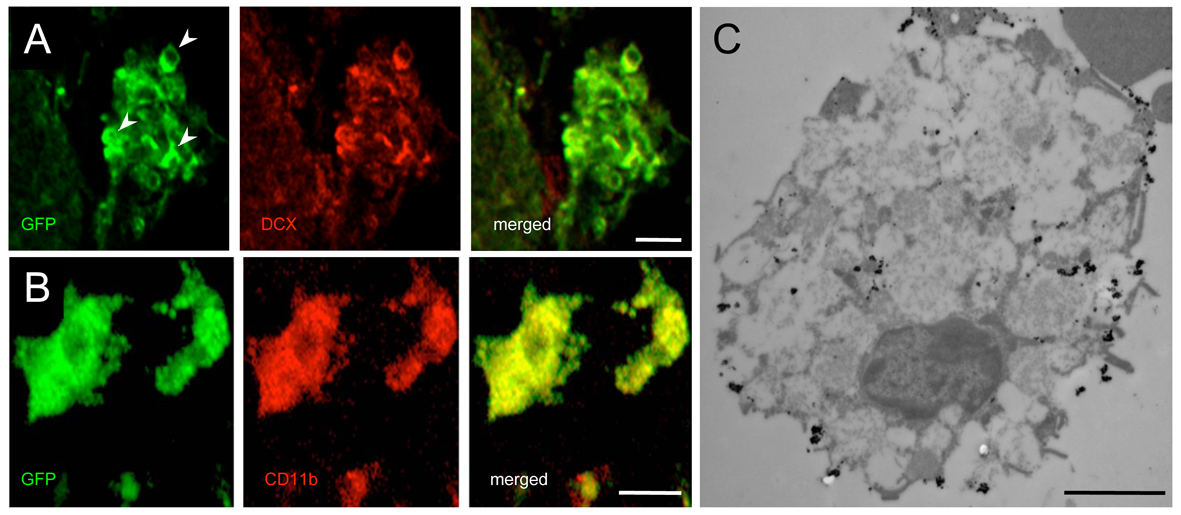

Supplement: Figure S2 — Phenotypical and morphological analysis of s.c.-injected NPCs accumulating into draining lymph nodes of R-EAE mice. A, Representative image of three s.c.-injected GFP+NPCs (arrowheads) co-expressing doublecortin (DCX) within a cervical lymph node. Scale bar: 40 µm. B, Representative image of two distinct lymph node CD11b+professional phagocytes being immune reactive also for GFP+. Scale bar: 10 µm. C, Transmission electron microscopy (TEM) of a vacuolized picnotic GFP+cell in a representative axillary lymph node. Note the presence of electron dense granules both in cytoplasm and cell surface. Scale bar: 2 µm. Images in A–C refer to representative draining lymph nodes from R-EAE mice injected s.c. with NPCs at 3 and 10 dpi and sacrificed at 72 days after cell injection. (2.07 MB TIF) [file pone.0005959.s002.tif]

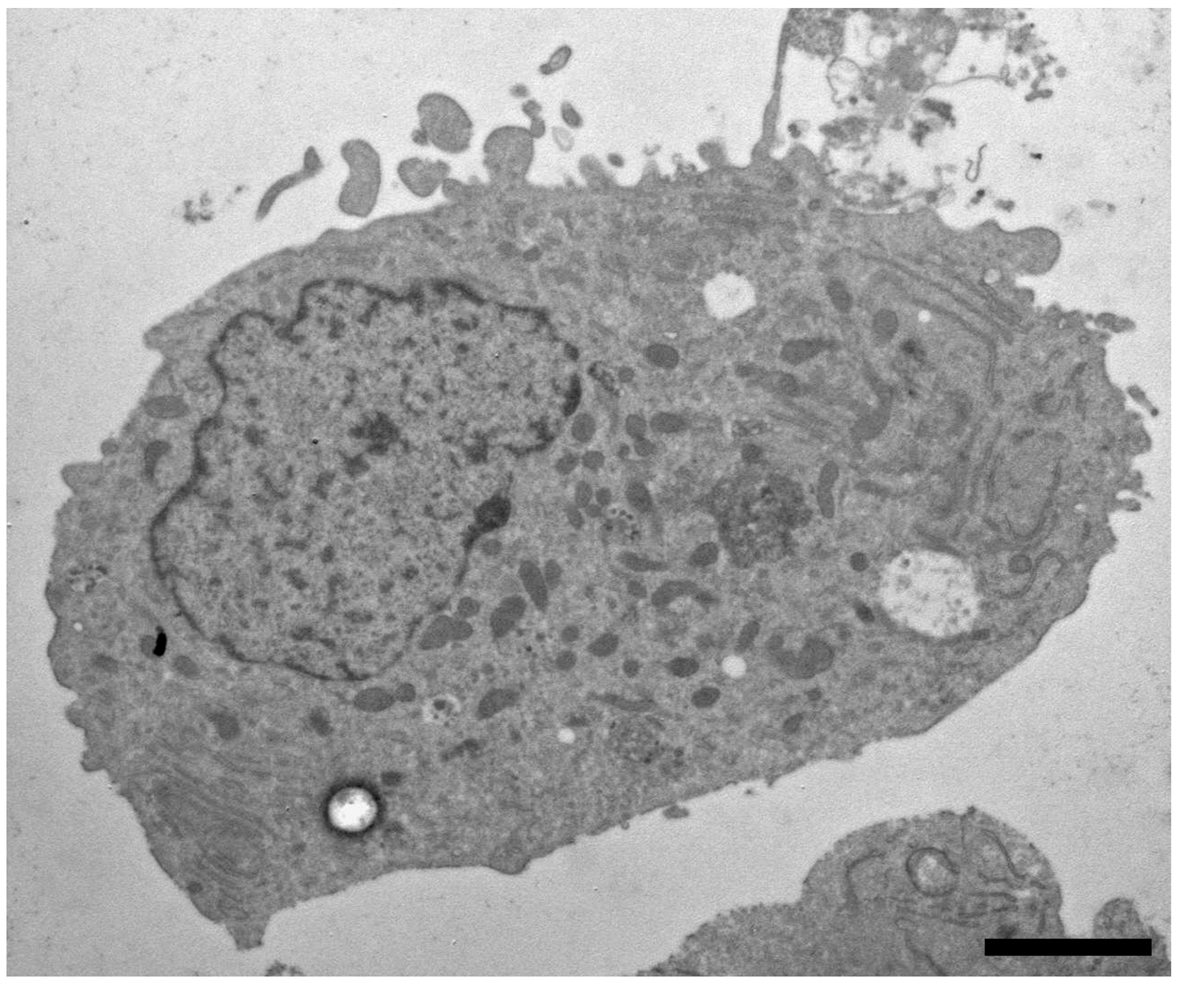

Supplement: Figure S3 — TEM image of a NPC from a neurosphere in vitro. Note the irregular nucleus, and the organelle-rich cytoplasm with abundant mitochondria and endoplasmic reticulum. Morphological and ultrastructural features of this single NPCs in vitro are similar to the NPCs detected in vivo in lymph nodes (see also Figure 2). Scale bar: 2 µm. (4.74 MB TIF) [file pone.0005959.s003.tif]

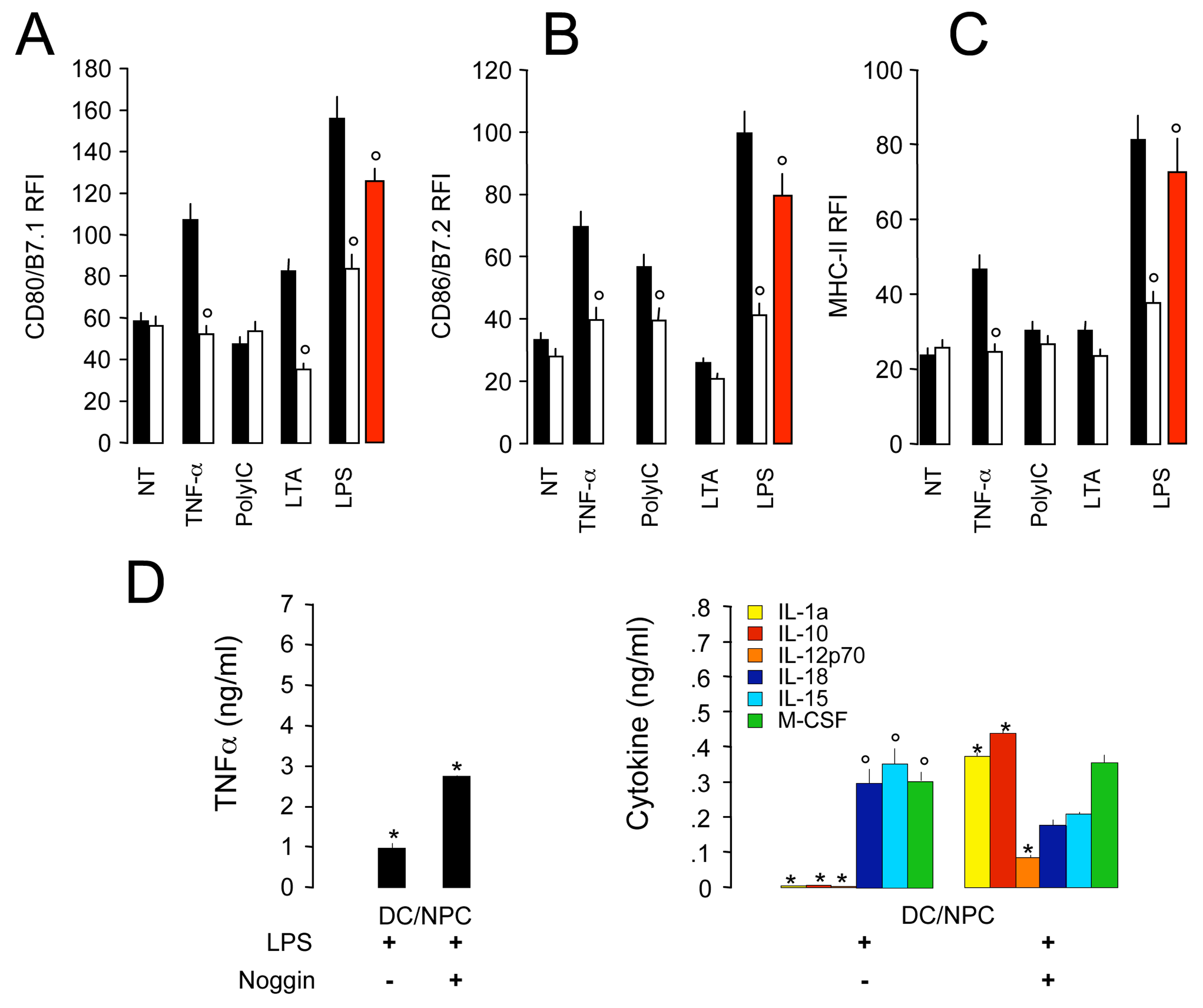

Supplement: Figure S4 — The BMP antagonist Noggin reverts the hindrance of DC maturation and cytokine production. A–C, Noggin (red bars) almost completely reverts the down-regulation of CD80/B7.1 (A), CD86/B7.2 (B), and MHC-II (C) obtained when DCs maturating in vitro with different TLR agonists are co-cultured in trans-wells with NPCs (white bars) (see also Figure 3). Black bars are non co-cultured control DCs, while NT are not treated DCs. Data are expressed as mean RFI over unstained (±SEM) from n>4 independent experiments. D, The addition of Noggin induces substantial recovery of cytokine levels, whose production is impaired in DC/NPC co-cultures. Data are mean cytokine levels (ng/ml) (±SEM) from a total of n>3 independent experiments. *p<0.05 and **p<0.005, vs. control DCs. (2.19 MB TIF) [file pone.0005959.s004.tif]

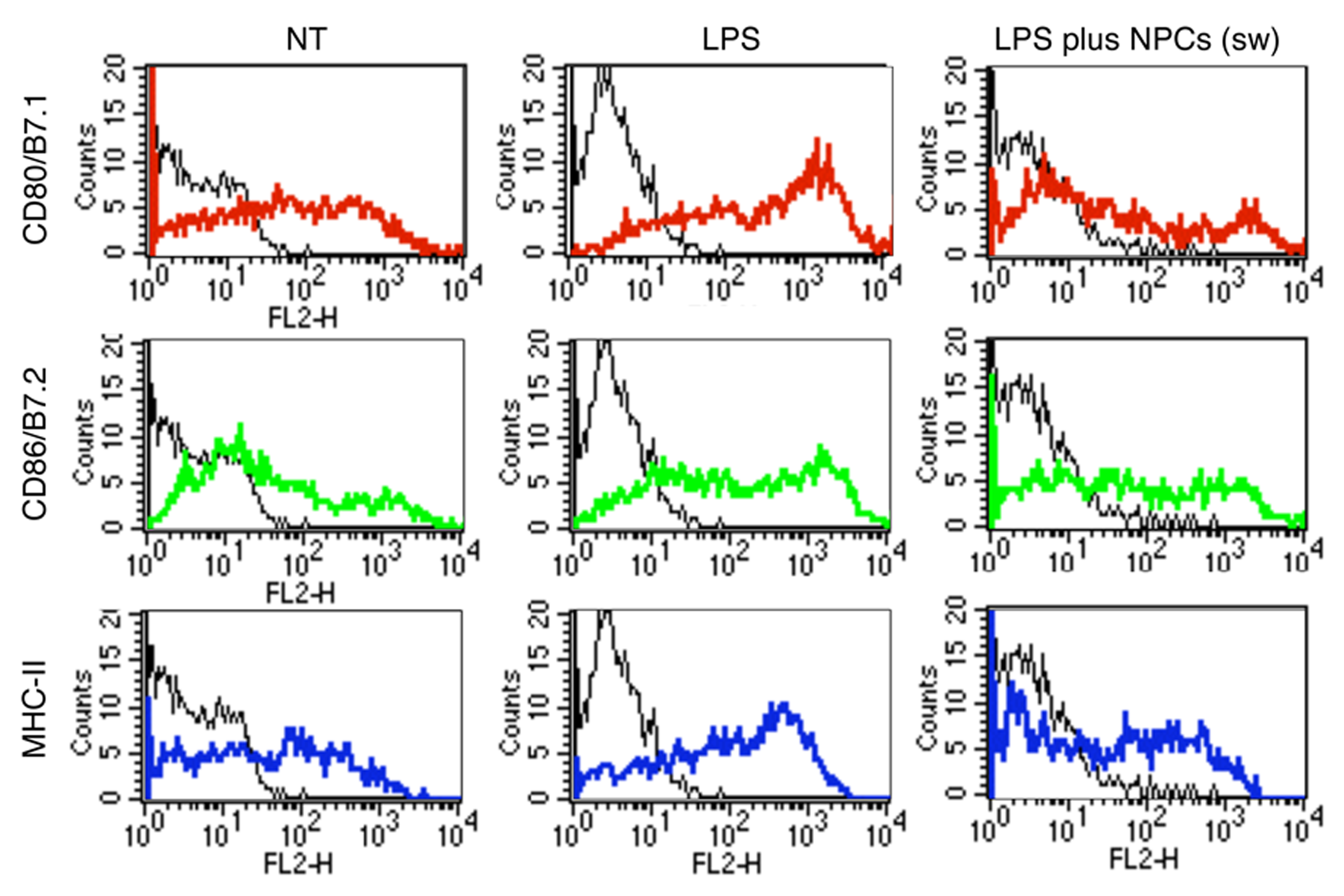

Supplement: Figure S5 — NPCs inhibit the up-regulation of co-stimulatory molecules upon LPS activation of DCs. Representative histograms showing the fluorescence intensity for CD80/B7.1 (red lines), CD86/B7.2 (green lines) and MHC-II (blue lines) on untreated (NT) DCs, DCs activated with LPS and DCs activated with LPS and co-cultured with NPCs in the same well. Black lines represent isotype controls. (8.69 MB TIF) [file pone.0005959.s005.tif]

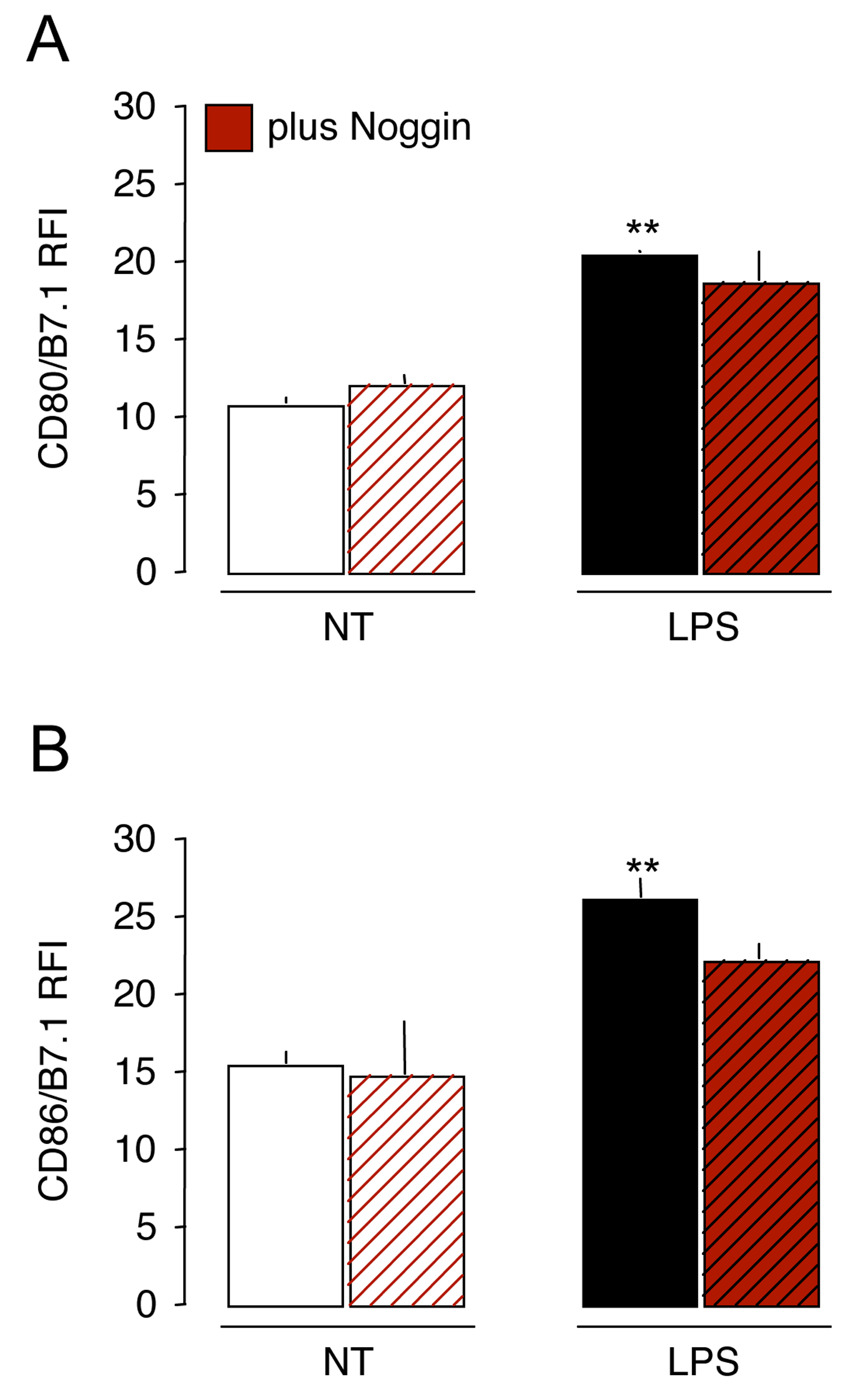

Supplement: Figure S6 — The BMP antagonist Noggin alone does not interfere with DC maturation in vitro. Soluble recombinant Noggin does not interfere with the expression of CD80/B7.1 and CD86/B7.2 onto DCs undergoing maturation with LPS in vitro. Data are mean RFI (±SEM) from a total of n = 2 independent experiments. **p<0.005, vs. control DCs. (1.45 MB TIF) [file pone.0005959.s006.tif]

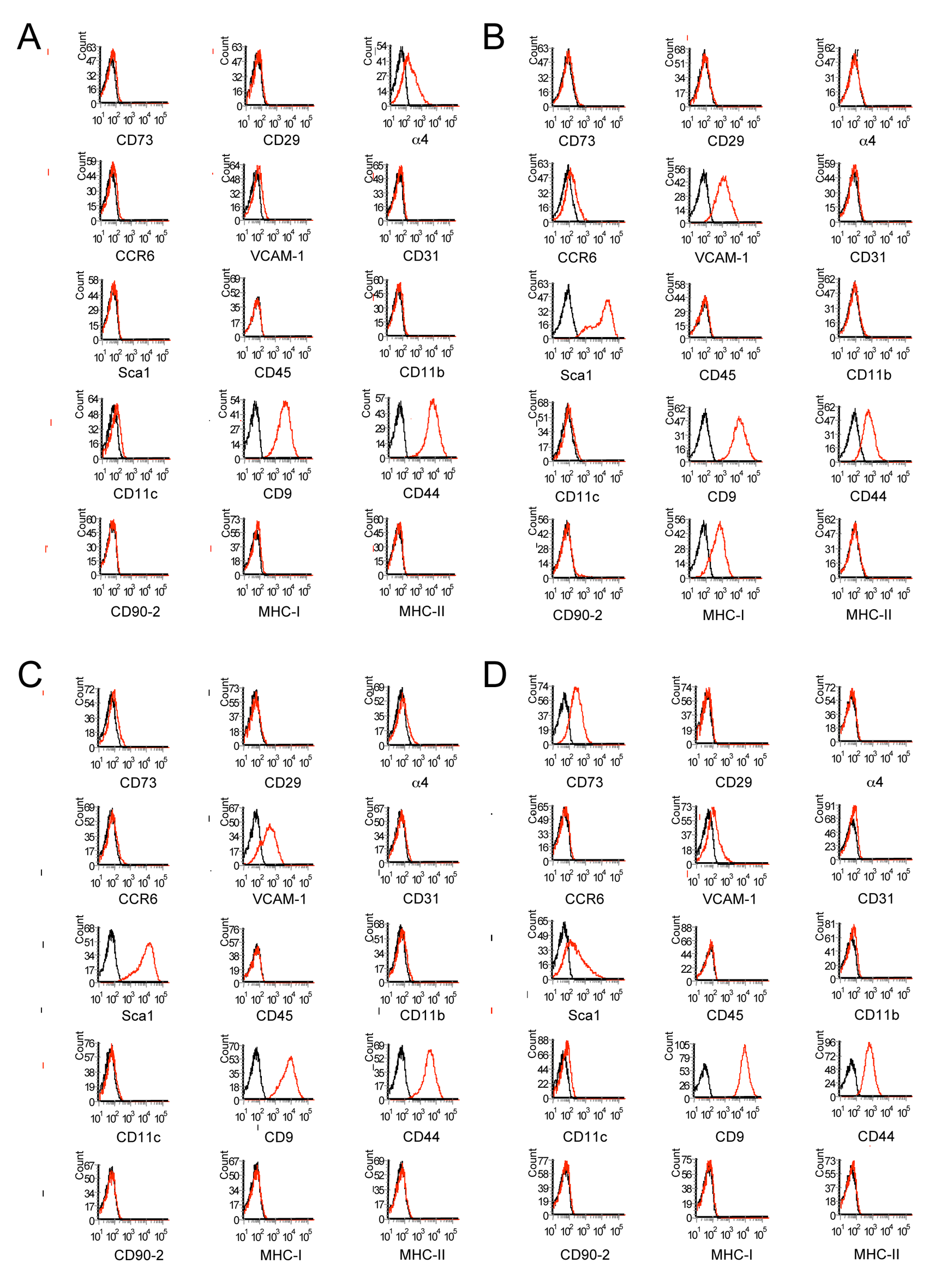

Supplement: Figure S7 — Phenotype of NPCs (A), bone marrow-derived MSCs (B), vessel-associated MSAs (C) and ATDC5 condrogenic cells (D). Histograms demonstrating the expression of surface molecules (red) are overlaid with unstained controls (black). (2.77 MB TIF) [file pone.0005959.s007.tif]
