# Supplementary material for: Immune Regulatory Neural Stem/Precursor Cells Protect from Central Nervous System Autoimmunity by Restraining Dendritic Cell Function
Source: PLoS One. 2009 Jun 19;4(6):e5959. doi: 10.1371/journal.pone.0005959 (PMC2694997; doi:10.1371/journal.pone.0005959)
Supplement: Material S1 — Supplementary Materials and Methods (0.08 MB DOC) [file pone.0005959.s008.doc]

**Supplementary Materials and Methods**

**NPC derivation and cultures**

Mice were anesthetized by intraperitoneal injection of pentobarbital (120 mg/kg) and killed by cervical dislocation. The brains were removed and placed in artificial cerebrospinal fluid (aCSF) (124 mM NaCl, 5 mM KCl, 1.3 mM MgCl2 , 0.1 mM CaCl2, 26 mM NaHCO3, and 10 mM D-glucose, pH 7.3) aerated with 95% O2/5% CO2 at room temperature. The SVZ neural tissue – excluding the subependyma – was isolated after coronal sectioning and cut into 1 mm^3^ pieces. Pieces were transferred into 30 ml of aCSF containing 1.3 mg/ml trypsin, 0.67 mg/ml hyaluronidase, and 0.2 mg/ml kynurenic acid (all from Sigma) and incubated, under continuous oxygenation and stirring, for 90 min at 32-34°C. Tissue sections were then rinsed in aCSF for 10 min, transferred to DMEM/F12 (Life Technologies) medium containing 0.7 mg/ml ovomucoid (Sigma), and carefully triturated with a fire-polished Pasteur pipette. Cells were collected by centrifugation and re-suspended in GF-free, chemically defined DMEM/F12 medium containing 2 mM L-glutamine, 0.6% glucose, 9.6 mg/ml putrescine, 6.3 ng/ml progesterone, 5.2 ng/ml sodium selenite, 0.025 mg/ml insulin, 0.1 mg/ml transferrin, and 2 μg/ml heparin (Sigma). Cells were then cultured in NeuroCult® Proliferation Kit (Stem Cell Technologies). The number of primary spheres was counted after 7-12 days in vitro (DIV). For cell amplification, 8000 cells/cm^2^ were plated at each sub-culturing passage in untreated tissue culture flasks. After 3-4 days (time estimated to obtain the doubling of cell number), neurospheres were harvested, mechanically dissociated, counted and re-plated under the same culture conditions. NPCs at passage number ≤ 15 were used in all experiments. For some in vitro experiments, single cell dissociated NPCs were fixed with 2% paraformaldehyde (PFA) for 5 minutes at room temperature and carefully washed (3 times) with PBS.

**EAE induction and NPC transplantation**

SJL mice (Charles-River) (n= 112 in total) were injected subcutaneously with 300 μl of 200 μg PLP139-151 (Espikem) in incomplete Freund’s adjuvant (IFA) containing 8 mg/ml of Mycobacterium tuberculosis (strain H37Ra; Difco). Body weight and clinical score (0= healthy; 1= limp tail; 2= ataxia and/or paresis of hindlimbs; 3= paralysis of hind limbs and/or paresis of forelimbs; 4= tetra paralysis; 5= moribund or death) were recorded daily. For NPC transplantation, single cell-dissociated NPCs were labelled *in vitro* using a third-generation lentiviral vector pRRLsin.PPT-PGK engineered with the *green fluorescent protein (GFP)* [1,2].

**In vitro expansion and adoptive transfer of PLP139-151-specific mouse T cells**

Lymph node cell (LNC) suspensions were prepared using 70 μm cell strainers (Becton Dickinson) and 5 x 10^6^ LNCs/ml were cultured for 4 DIV at 37°C and 7% CO_2_ in 5 ml of RPMI (Gibco) supplemented with 50 μM 2-β-mercaptoethanol (Gibco), 2 mM L-glutamine (BioWhittaker), 1 mM Sodium Piruvate (BioWhittaker), 1 mM penicillin (Gibco), 100 μg/ml streptomycin (Gibco), and 10% FCS (Sigma) (*RPMI complete medium*), in presence of 30 μg/ml PLP139-151 (Espikem). During the following 10 DIV, the cells were allowed to proliferate and then challenged for 4 additional DIV with 30 μg/ml PLP139-151 in presence of irradiated spleen-derived antigen presenting cells (APC) at a 1:5 T cell/APC ratio. At the end of this second challenge, LNCs were characterized for either Th1 or Th2 cytokine production as either IFN-β+/TNF-α+/IL-4^-^ or IFN-γ-/TNF-α-/IL-4^+^, respectively, by using intracellular staining followed by FACS analysis.

For adoptive EAE transfer, LNCs were prepared from PLP139-151-immunized mice and co-cultured with syngenic NPCs. Then, following the first challenge with PLP139-151, 10^6^ purified PLP-specific T cells were injected intravenously in 6-8 week-old female naïve SJL mice. Body weight and clinical score were monitored in adoptively transferred EAE as described above for active immunization.

For ex vivo proliferation assays, axillary, cervical and inguinal lymph nodes from each mouse were surgically removed at 10 dpi. Single LNC suspensions were prepared and a total of 5 x 10^5^ cells/200 μl of stimulation media [HL1(Biowhittaker), 2% ultraglutamine (Biowhittaker), 50 μg/ml gentamycin (Sigma)] plated on a 96-well, round bottom tissue culture plate without (control) or with the nominal antigen (3-10 μg/ml PLP139-151) and then incubated at 37° C for 72 hours. The LNCs were then pulsed with 1 μCi [^3^H] methylthymidine for the final 18 hours. Cells were finally harvested on filters and methylthymidine uptake measured using a liquid scintillation counter. Proliferation was determined from triplicate cultures and the data were expressed as the mean proliferation index ratio (± SEM) between the total counts per minute (cpm) of each given sample and the cpm of controls.

**Dendritic cell (DC) preparation and co-cultures**

[*Continues from main text*] When indicated, DCs were activated at day 5 for further 48 hours with TNF-α (100 ng/ml, R&D system) or TLR agonists, such as bacterial LPS (1μg/ml, *Escherichia coli* serotype 026:B6, Sigma Aldrich), poly-IC (20 µg/ml, Amersham Biosciences), LTA (4 μg/ml, Amersham Biosciences). For some experiments, DCs were induced to mature with LPS in the presence of recombinant human BMP-4 (0.5 ng/ml, R&D system), mouse recombinant Noggin (100 ng/ml, R&D Systems), recombinant mouse Shh (0.5 ng/ml, R&D System) and recombinant mouse Cyclopamine (1 μM, R&D System).

Co-cultures were performed either in presence or in the absence of LPS, as indicated above. In order to agonize BMP-4, mouse recombinant Noggin (see above), was added at day 5 to co-cultures with or without LPS. At day 7, DCs were retrieved and analysed for FACS-activated surface staining and cytokine production on supernatants. In the experiments aimed at clarify the persistence of the NPC-mediated effect on DCs, after 24 hours of co-culture in the presence of LPS, NPCs were removed from the co-culture system, DCs (both co-cultured as well as controls) washed with PBS and re-plated in their own maturating medium (see above) supplemented with LPS.

To verify the effect of NPCs on PLP139-151 antigen presentation, immature DCs or DCs stimulated with LPS – alone or in combination with NPCs and/or Noggin (see above) – were cultured *in vitro* for 18 hours in the presence of PLP139-151 (20 μg/ml). Antigen-pulsed DCs were then cultured with syngeneic CD4^+^ T cells derived from mice immunized 16 days before with PLP139-151 in CFA. T cells were analyzed as above. DCs propagated in vitro were analyzed for gene expression either immature or after incubation for the last 48 hours of culture in the presence of LPS. Further information is provided as Material S1.

**MSC isolation and long-term culture**

Total bone marrow was collected from 7-12 week-old female SJL/J mice (Charles River) by flushing femurs and tibias with HBSS, as previously described [3]. For mesenchymal stem cell (MSC) cultures, the total bone marrow was resuspended and plated in uncoated plastic dishes in MesenCult Basal Media supplemented with MSC stimulatory supplements (StemCell Technologies). Cultures were expanded in 5% oxygen until cultures reached 80% confluency and serially passaged with 0.05%/0.02% (w/v) trypsin/EDTA (Gibco). Cell characterization and phenotype was performed by FACS at time of co-cultures (Figure S7). MSCs at passage number ≥ 8 were used in all experiments.

**MSA long-term culture**

Vessel-associated mesoangioblasts (MSAs) were kindly provided by Giulio Cossu and expanded as previously described [4]. Briefly MSAs were cultured in high-glucose DMEM supplemented with 20% (v/v) foetal bovine serum (FBC), 2 mM glutamine, 1 mM penicillin and 100 μg/ml streptomycin. Cells were sub-cultured using trypsin/EDTA (all from Gibco), and maintained at 37°C, 5% CO_2_, 5% O_2_, 90% N_2_ in a humidified incubator. Cell characterization and phenotype was performed by FACS at time of co-cultures (Figure S7). MSAs at passage number ≥ 8 were used in all experiments.

**ATDC5 mouse embryonic condrogenic progenitor cells**

For routine expansion, the ATDC5 mouse embryonic condrogenic progenitor cell line (Riken Cell Bank) was cultured in a 1:1 mixture of DMEM/F12, containing 5% FBS, 2 mM glutamine, 1 mM penicillin, 100 μg/ml streptomycin (all from Gibco), as previously described [5]. Cells were sub-cultured using trypsin/EDTA (both from Gibco), and maintained at 37°C in a humidified atmosphere of 5% CO_2_ in air. Cell characterization and phenotype was performed by FACS at time of co-cultures (Figure S7).

**NIH 3T3 mouse embryonic fibroblast cells**

For routine expansion, the NIH 3T3 mouse embryonic fibroblast cell line was expanded in complete Iscove’s Medium (IMDM), containing 10% FBC, 2 mM glutamine, 1 mM penicillin, 100 μg/ml streptomycin, as previously described [6]. Cells were sub-cultured using trypsin/EDTA (both from Gibco), and maintained at 37°C in a humidified atmosphere of 5% CO_2_ in air.

**RT-PCR**

Cell samples for relative quantification were collected. Two millions cells for each sample were lysed in lysis buffer (Qiagen) and stored at – 80°C until the RNA was extracted following manufacture’s instructions. Residual genomic DNA was removed by incubating with DNase I, RNase-free (Qiagen) and eluted from the RNeasy mini columns with RNase-free water. The amount of total RNA was quantified using a NanoDrop ND-100 (Nano Drop Technologies) and the cDNA was synthesized from 5 μg of total RNA using the Ready-to-Go kit (Amersham) and a mixture of random examers (pd(N)_6_) used as primer. After purification using QIAquick PCR Purification kit (Qiagen), the cDNA samples were diluted 1:6 prior use in QPCR. Twenty-five ng of cDNA were used for Real-time PCR using pre-developed Taqman Assay Reagents (Applied Biosystems). Real-time quantitative PCR was performed with an ABI Prism™ 7700 Sequence Detection System (Applied Biosystems) according to manufacturers protocol. We used the housekeeping gene, GAPDH, as normalizer and ConA (5 mg/ml for 72 hours) -activated splenocytes (from strain-, sex- and age-matched mice) as internal calibrator. Data were generated using bone marrow-derived SJL DCs (NT and LPS-treated, see above) and NPCs at ≤10 passages of in vitro amplification. Results are expressed as mRNA arbitrary units (AU) from n= 3 independent experiments and represent the mean fold induction (± SEM) over NPCs.

**Western blot analysis**

Lymph node sampling was performed at two weeks after sham/NPC treatment from mice transplanted s.c. with NPCs at 3 and 10 dpi. Frozen tissue samples were homogenated using lysis buffer (20 mM TRIS.HCl pH 7.4, 260 mM Sucrose) with the addition of mixed protease inhibitors, on ice. Supernatants were collected after centrifugation at 14,000 rpm for 10 min at 4°C. Protein concentration was determined by the Bradford method. Protein samples (60-80 μg), prepared in a final 1X Laemmli Buffer, were denatured for 5 min at 100°C and resolved on 12% (Noggin) and 15% (BMP-4 and Shh) SDS-polyacrylamide minigels. After electrophoresis, proteins were transferred to either polyvinylidene difluoride (PVDF) (Noggin) or nitrocellulose filters (BMP-4 and Shh). Filters were subsequently blocked by soaking, at room temperature, the blots in 1X TBS (Tris Buffered Saline), 5% powdered fat-free milk and 0.1 % Tween-20 for 1 hour, followed by incubation with rat anti-Noggin (R & D Systems), mouse anti-BMP-4 (Chemicon) and rabbit anti-BMP-4 (Abcam), goat anti-Shh (R & D Systems) and with mouse anti β-actin (Sigma) in blocking buffer.

Analysis of ERK1/2, phospho ERK1/2, p38, phospho p38, SMAD 1 and phospho-SMAD1/5/8 proteins expression was performed using untreated and LPS-treated dendritic cells. LPS-treated DCs (1 μg/ml) were collected at 30’ after treatment in vitro w/wo BMP4 (0.5 ng/ml) or BMP-4 + Noggin (0.5 ng/ml and 100 ng/ml, respectively), chilled on ice, and lysed using Cell Lysis Buffer (Cell Signaling, #9803) containing 1X PMSF and mixed protease inhibitors, added just prior to use. Supernatants were collected after centrifugation at 14,000 rpm for 10 min at 4°C. Proteins concentration was determinated by the Bradford method. Protein samples (80 μg), prepared in a final 1 X Laemmli Buffer, were denatured for 5 min at 100°C and resolved on 12% SDS-polyacrylamide minigels. After electrophoresis, proteins were transferred to nitrocellulose filters. Filters were subsequently blocked by soaking, at room temperature, in 1 x TBS (Tris Buffered Saline), 5% powdered non fat milk and 0.1 % Tween-20 for 1 hour, followed by incubation with mouse anti Erk1/2 (Abcam, # ab 36991), mouse anti phospho Erk1/2 (Abcam, # 50011), mouse anti p38 (Abcam, # ab 31828), rabbit anti phospho p38 (Abcam, # ab 4822), rabbit anti SMAD1 (Abcam, # ab 63356), rabbit anti phospho Smad 1/5/8 (Cell Signaling, # 9511), Rabbit anti Smad 4 antibody (Cell Signaling cat. 9515) and with mouse anti-β actin (Sigma, # A5441) antibodies in blocking buffer.

After washing, blots were incubated with horseradish peroxidase conjugated anti-mouse, anti-goat or anti-rabbit IgG secondary antibodies in blocking buffer for 1 hour at room temperature and rinsed. Secondary antibody bindings were detected by reaction with Supersignal West Dura Extended Duration Substrate (Pierce, #34075) for 5 min at room temperature before exposing the blots to X-ray film. Intensities of all bands on immunoblots were quantified by densitometry (ImageQuant software, Molecular Dynamics). Single band densitometry values were normalized over β-actin and data were expressed as protein arbitrary units (AU) (Fold Induction over average naïve) (± SEM).

**FACS analysis**

LNCs were stained with anti CD4, CD27, CD44 and CD62L (all from BD Pharmingen). Intracellular cytokine staining was performed by stimulating cells with ionomycin and phorbol 12-myristate 13-acetate (PMA; both 1 μg/ml, Sigma), in the presence of Brefeldin A (1 μg/ml, Sigma), for 5 hours at 37°C and 5% CO_2_. Anti-IFN-γ, IL-4, IL-2, and IL-10 were obtained from BD Pharmingen. DCs were stained with anti-CD11c (clone HL3), -CD86 (clone GL1), -CD80 (clone 16-10A1), -CD40 (clone 3/23, all from BD) or -MHC class II (clone M5/114.15.2, Cedarlaine).

NPCs, MSCs, MSAs and ATDC5 cells (0.2 X 10^6^ cells/condition) were stained with anti CD31 (Clone MEC 13.3), CD34 (Clone RAM34), CD45 (clone 30-F11), -CD44 (clone IM7), Sca-1/Ly-6A/E (Clone E13-161.7), CD11b (Clone M1/70), -CD11c (Clone HL3), CD90.2 (Thy1.2) (Clone 53-2.1), CD73 (Clone TY/23) (all from BD Biosciences); H-2KdDd (Class I) (Clone 34-1-2S), RT1.Bu (Clone OX-3), CD106 (VCAM) (Clone MK2) (from Cedarlane); CCR6 (Clone 140706) (from R&D); alpha 4 integrin (VLA4) (Clone PS/2) (from Abcam); CD9 (Clone KMC8) (from eBioscience); CD29 (Clone MEM-101A) from Immunotools. Isotype-matched control antibodies were included in each staining.

**Tissue pathology**

Paraffin embedded 5-7 μm-tick spinal cord sections were stained with haematoxilin and eosin, Luxol fast blue and Bielshowsky to detect inflammatory infiltrates, demyelination and axonal loss, respectively [1]. Light microscopy (Olympus, BX51) was performed on a total of n≥ 20 spinal cord sections/mouse (n= 3 mice/group) and quantification of CNS damage was performed, using IM-50 image analyser software (Leica).

To detect in vivo transplanted NPCs, 10-12 μm-tick frozen tissue sections were cut and processed for immunofluorescence for GFP. The following primary antibodies were used: rabbit anti-GFP (Molecular Probes), goat anti-BMP-4 (1:100, Santa Cruz Biotechnology), rat-anti tenascin C (1:100, Abcam), goat anti-Sonic Hedgehog (1:50, R&D Systems), rat anti-F4/80 (1:200, Abcam), rabbit anti-Von Willebrand Factor (1:1000, Abcam), mouse anti-rat RT1.B (MHC-II, 1:100, Cedarlane), rat anti-mouse CD45 (1:100, BD Biosciences), rat anti-mouse Embryonic NCAM (CD56) (1:100, BD Biosciences), rabbit anti-neuronal class III β–Tubulin (1:1000, Covance), mouse anti neuronal nuclei (NeuN) (1:1000, Chemicon), rabbit anti-NG2 (1:100, Chemicon), rabbit anti-GFAP (1:500, Dako), rabbit anti-PDGFr α (1:1000, Santa Cruz Biotechnology). Nuclei were stained with 4'-6-diamidino-2-phenylindole (DAPI) (Roche). Appropriate anti-rat, -mouse, -goat and -rabbit fluorophore- (Alexa-fluor 488, 546; Molecular Probes) or biotin- (Amersham) conjugated secondary antibodies were used. Lymph nodes from naïve, sex-, strain-, age- and weight-matched SJL mice (n= 3) were used as controls for in vivo detection of stem cell regulators. Confocal microscopy (Leica DM 6000B) was performed on 15 to 50 μm-tick frozen brain, spinal cord and lymph node tissue sections obtained from n= 3 EAE mice injected s.c. with NPCs (both 3 and 10 dpi and 10 dpi only). Tissue sections processed for confocal imaging were analyzed at 0.5 μm intervals.

**Immunogold and electron microscopy**

Transverse 50 μm-tick free-floating node sections were cut on a vibratome. Following incubation in blocking mix (PBS 1X; BSAC 0.3%), primary chicken anti-GFP antibody (1:200, Aves Labs.) was added and incubated at 4°C for 4 days. Secondary antibodies conjugated to colloidal gold (0.8 nm) were then applied for 24 hours at room temperature. The following day, sections were washed in PBS and in sodium acetate. The signal enhanced by immersion in A and B solution (Aurion). Sections were then incubated in 0.05% gold chloride and with 0.3% sodium thiosulfate. Then, sections were incubated in 2% glutaraldehide. Immunostained sections were post-fixed in 1% osmium, rinsed, dehydrated, and embedded in araldite (Durcupan, Fluka). 1.5 μm-thick semithin sections of selected lymph node areas showing gold staining were cut with a diamond knife. Labelled cells were analyzed on n= 4 mice taking off the semithins and cutting ultrathin (70 nm) sections. For the ultrastructural analysis of NPCs previous to transplantation, floating neurospheres were fixed with 3.5 % glutaraldehide and embedded in 2 % agar. NPCs were then rinsed, dehydrated, embedded in araldite and processed for conventional EM. Tissue sections and NPCs were studied under a Fei Tecnai Spirit Electron Microscope.

**Hystochemistry**

Free-floating node sections were rinsed with potassium phosphate-buffered saline (KPBS) and pre-incubated in 5% of appropriate sera (e.g., normal donkey serum for DCX) in KPBS containing 0.25% Triton-X. Sections were then incubated with either goat anti-DCX (1:100, Santa Cruz Biotechnology), rabbit anti-nestin (1:150, Sigma) or allophycocyanin (APC)-conjugated rat anti-CD11b (1:150, Abcam) in pre-incubation solution at 4°C. Sections were then rinsed with KPBS containing 0.25% Triton-X, followed by incubation with Texas Red-conjugated appropriate donkey anti-goat secondary antibody (1:200, Jackson ImmunoResearch Laboratories Inc) for visualization of DCX or Alexa 555 for nestin. Nuclei were visualized with DAPI. The sections were mounted onto glass slides with Fluorsave mounting medium. The slides were studied in the Leica SP2 confocal microscope. In order to analyze cell distribution throughout the whole lymph node, n≥ 8-10 confocal microscopy images per lymph node were taken at 10X magnification on 50 μm-tick vibratome node sections from n= 4 mice showing GFP fluorescence as well as both DCX and CD11b immunoreactivity and then mounted with Adobe Photoshop.

**References**

1. Pluchino S, Quattrini A, Brambilla E, Gritti A, Salani G, et al. (2003) Injection of adult neurospheres induces recovery in a chronic model of multiple sclerosis. Nature 422: 688-694.

2. Follenzi A, Ailles LE, Bakovic S, Geuna M, Naldini L (2000) Gene transfer by lentiviral vectors is limited by nuclear translocation and rescued by HIV-1 pol sequences. Nat Genet 25: 217-222.

3. Sung JH, Yang HM, Park JB, Choi GS, Joh JW, et al. (2008) Isolation and characterization of mouse mesenchymal stem cells. Transplant Proc 40: 2649-2654.

4. Tonlorenzi R, Dellavalle A, Schnapp E, Cossu G, Sampaolesi M (2007) Isolation and characterization of mesoangioblasts from mouse, dog, and human tissues. Curr Protoc Stem Cell Biol Chapter 2: Unit 2B 1.

5. Atsumi T, Miwa Y, Kimata K, Ikawa Y (1990) A chondrogenic cell line derived from a differentiating culture of AT805 teratocarcinoma cells. Cell Differ Dev 30: 109-116.

6. Jainchill JL, Aaronson SA, Todaro GJ (1969) Murine sarcoma and leukemia viruses: assay using clonal lines of contact-inhibited mouse cells. J Virol 4: 549-553.
